# Mitochondrial Dysfunction Plus High-Sugar Diet Provokes a Metabolic Crisis That Inhibits Growth

**DOI:** 10.1371/journal.pone.0145836

**Published:** 2016-01-26

**Authors:** Esko Kemppainen, Jack George, Görkem Garipler, Tea Tuomela, Essi Kiviranta, Tomoyoshi Soga, Cory D. Dunn, Howard T. Jacobs

**Affiliations:** 1 BioMediTech and Tampere University Hospital, FI-33014, University of Tampere, Tampere, Finland; 2 Department of Molecular Biology and Genetics, Koç University, Sariyer, Istanbul, 34450, Turkey; 3 Institute for Advanced Biosciences, Keio University, Tsuruoka, Yamagata, 997–0035, Japan; 4 Institute of Biotechnology, FI-00014, University of Helsinki, Helsinki, Finland; CINVESTAV-IPN, MEXICO

## Abstract

The *Drosophila* mutant *tko*^*25t*^ exhibits a deficiency of mitochondrial protein synthesis, leading to a global insufficiency of respiration and oxidative phosphorylation. This entrains an organismal phenotype of developmental delay and sensitivity to seizures induced by mechanical stress. We found that the mutant phenotype is exacerbated in a dose-dependent fashion by high dietary sugar levels. *tko*^*25t*^ larvae were found to exhibit severe metabolic abnormalities that were further accentuated by high-sugar diet. These include elevated pyruvate and lactate, decreased ATP and NADPH. Dietary pyruvate or lactate supplementation phenocopied the effects of high sugar. Based on tissue-specific rescue, the crucial tissue in which this metabolic crisis initiates is the gut. It is accompanied by down-regulation of the apparatus of cytosolic protein synthesis and secretion at both the RNA and post-translational levels, including a novel regulation of S6 kinase at the protein level.

## Introduction

Mitochondrial DNA encodes just 13 polypeptides in most metazoans, representing a small but vital subset of the subunits of the apparatus of oxidative phosphorylation (OXPHOS). In addition it encodes the RNA components, i.e. 2 rRNAs and 22 tRNAs, that contribute to the separate machinery of protein synthesis inside mitochondria, that generates these polypeptides. Defects in mitochondrial protein synthesis are a frequent primary cause of mitochondrial disease: see reviews [1, 2]. Their genetic origin can be either nuclear or mitochondrial, and they may affect mitochondrial rRNAs, tRNAs, ribosomal proteins, translation factors, aminoacyl-tRNA synthetases, tRNA modifying enzymes or other accessory factors. They can show a wide range of tissue-specific symptoms that remains largely unexplained, varying dramatically also in severity and age of onset. Whilst the consequences of the primary molecular defect may be clear at the level of the translational machinery, the link to pathogenesis is poorly understood. It is assumed to have metabolic, cellular, physiological and developmental dimensions, the elucidation of which requires well-controlled studies in appropriate model systems.

Our analyses have focused on a *Drosophila* model of mitochondrial disease (*tko*^*25t*^) [3] in which a nuclear mis-sense mutation in the gene for mitoribosomal protein S12 [4] gives rise to a quantitative defect in mitochondrial protein synthesis [5] impacting all four OXPHOS enzyme complexes dependent on mitochondrial translation products [6]. The molecular phenotype of *tko*^*25t*^ resembles that seen in human disorders resulting from point mutations in proteins of the mitoribosomal small subunit, including MRPS16 [7] and MRPS22 [8]. At organismal level the *tko*^*25t*^ mutation gives rise to a complex developmental and behavioral phenotype, whose main features are larval developmental delay and susceptibility to paralytic seizures induced by mechanical shock, described as bang-sensitivity [6]. *tko*^*25t*^ adults show an altered pattern of gene expression [9] that suggests a transformation of metabolism to accommodate the OXPHOS defect engendered by the mutation. Specifically, there is up-regulation of lactate dehydrogenase and of enzymes involved in the dietary mobilization and catabolism of protein and fat that suggest a switch to glycolysis for ATP production and to other substrates for the supply of carbon skeletons for biosynthesis.

The phenotype of developmental delay and bang-sensitivity, as well as the main (bisexual) changes in gene expression in adults, are shared with the mutant strain *sesB*^*1*^ [10], carrying a point mutation in the gene encoding the major isoform of the adenine nucleotide translocase [11]. Since the *sesB*^*1*^ mutation limits the supply of mitochondrially supplied ATP to the cell, we infer that these common features are the signature of defective OXPHOS in *Drosophila*. Furthermore, the *tko*^*25t*^ phenotype is not alleviated by expression of the non proton-motive alternative oxidase AOX from *Ciona intestinalis*, nor by the non proton-motive alternative NADH dehydrogenase Ndi1 from yeast [12], despite the fact that AOX and Ndi1 can partially compensate for the specific phenotypes produced by knockdown of subunits of OXPHOS complexes IV and I, respectively [9, 13, 14]. This supports the idea that the developmental phenotype of *tko*^*25t*^ results primarily from insufficient mitochondrial ATP production, rather than disturbed redox homeostasis.

The *tko*^*25t*^ phenotype can be partially alleviated by genetic suppressors in either nuclear or mitochondrial DNA [15, 16], which appear to increase the supply of mitochondrial translation products above a certain threshold. These findings suggested that we might also be able to manipulate the phenotype environmentally, by culturing the flies on media containing a different balance of dietary components. Specifically, we reasoned that media rich in sugars should support glycolysis and thus alleviate the mutant phenotype. If successful this could suggest a potential route to dietary management of patients with mitochondrial disease, particularly those with a global impairment of mitochondrial protein synthesis and thus of OXPHOS.

Surprisingly we found an opposite effect, namely that the *tko*^*25t*^ phenotype was exacerbated by sugar-rich media. Analysis of metabolite levels, gene expression, drug sensitivity and the status of proteins known to be involved in growth regulation and metabolite sensing has allowed us to construct a profile of the metabolic changes produced by the combination of mitochondrial dysfunction and high sugar diet, and a mechanistic model for how this leads to severe growth impairment. This knowledge has broad implications for understanding disease processes and their relation to diet.

## Results

### High-sugar diet exacerbates the growth-retardation of flies with mitochondrial dysfunction

**Fig 1:**
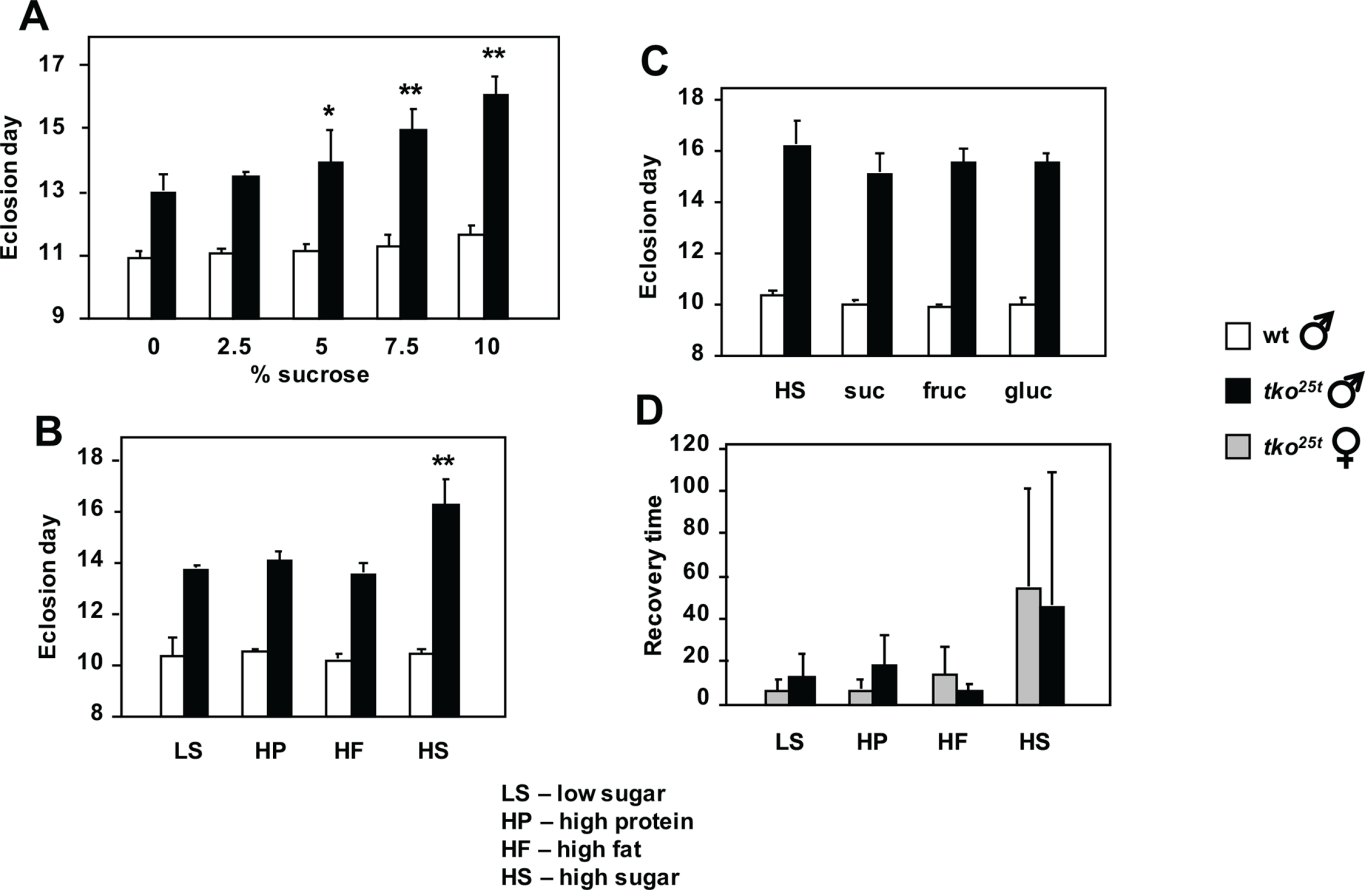
Modulation of *tko*^*25t*^ phenotype by diet. (A-C) Time to eclosion and (D) recovery time from mechanical shock (bang-sensitivity) of *tko*^*25t*^ and wild-type flies grown on media of the indicated composition (see SI for details). In (A) asterisks denote data classes significantly different from flies of the same genotype grown on 0% sucrose medium (Student’s *t* test, * showing *p* < 0.05, ** showing *p* < 0.01). In (B) asterisks (**) denote significant difference from flies of the same genotype grown on all other media tested (Student’s *t* test, *p* < 0.01), which were not significantly different from each other. In (C) there were no significant differences from flies of the same genotype, grown on other media (Student’s *t* test, *p* > 0.05). In all experiments eclosion times for *tko*^*25t*^ flies were also significantly different from those of wild-type flies grown on the same medium (Student’s *t* test, *p* < 0.01). In (D), the wide variance inherent to the phenotype precludes a standard statistical analysis. For corresponding eclosion data of females see Panel A in S1 Fig.

In order to test whether sugar supplementation would mitigate the developmental retardation fsof *tko*^*25t*^ flies, we cultured flies on different media containing a standard yeast composition (3.5% dried yeast, w/v), but increasing amounts of sucrose. Surprisingly, whilst the wild-type control strain was only minimally affected by the amount of sugar in the food, *tko*^*25t*^ flies developed more slowly on media with increasing sucrose concentration (Fig 1A and Panel A in S1 Fig). Males (Fig 1A) and females (Panel A in S1 Fig) were similarly affected, and the highest sucrose concentration tested (10%) doubled the developmental delay from ~2 to ~4 days at 25°C.

Because the high-sugar diets tested contain a higher caloric content than the low-sugar diets, we next tested isocaloric diets in which sugar was replaced with additional protein or fat (or a mixture). Once again, high-sugar diet exacerbated the developmental delay of *tko*^*25t*^ flies, but the proportion of fat and protein as substitute calories made no difference, and the two sexes behaved similarly (Fig 1B and Panel C in S1 Fig). Wild-type flies developed similarly on all diets tested. The specific sugar present in the food was also immaterial: there was no significant difference in the developmental delay of *tko*^*25t*^ flies grown on diets containing only sucrose, fructose or glucose, compared with the standard mixed-sugar diet (Fig 1C and Panel D in S1 Fig). The bang-sensitivity of *tko*^*25t*^ adults of both sexes was also alleviated by development on diets containing lower sugar content (Fig 1D). Wild-type flies were not bang-sensitive on any diet tested.

### Fly larvae with mitochondrial dysfunction exhibit a global anti-sugar response

In previous transcriptomic analyses [9] we noted that *tko*^*25t*^ adults manifested altered expression of some specific sugar transporters in the gut and Malpighian tubule (the equivalent of the mammalian kidney). Because of the unexpected observation that excess sugar is deleterious to *tko*^*25t*^ adults, we analyzed the expression of these genes, for clues to the mechanisms of sugar-responsiveness of *tko*^*25t*^.

**Fig 2:**
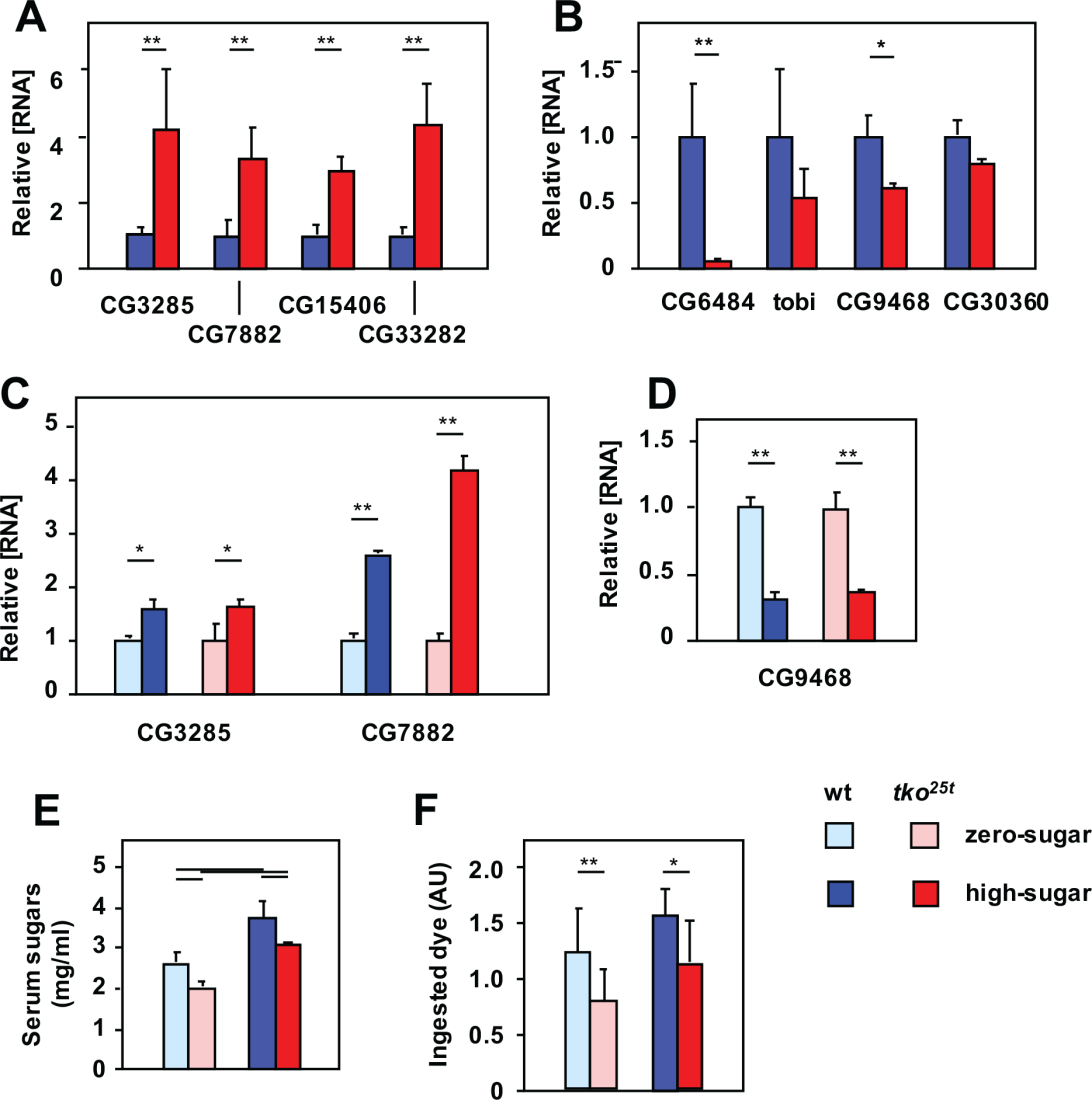
*tko*^*25t*^ flies manifest an ‘anti-sugar’ response. (A-D) Expression levels of various genes, based on QRTPCR, in L3 larvae of the indicated genotypes and growth conditions. (A, C) Malpighian tubule-specific sugar transporters, (B, D) gut-specific α-glucosidases; (A, B) all signals normalized to the levels in wild-type larvae, (C, D) all signals normalized to larvae of the same genotype grown on zero-sugar medium (i.e., ignoring the differences between genotypes). All values are significantly different between genotypes or diets, as plotted (Student’s *t* test, *p* < 0.01). (E) Total serum sugar concentrations in larvae of the indicated genotypes and growth conditions. Horizontal bars denote significantly different data classes (Student’s *t* test, *p* < 0.01). (F) Larval feeding rate, based on dye ingestion assay, in larvae of the indicated genotypes and growth conditions. Horizontal bars denote significantly different data classes (Student’s *t* test, * showing *p* < 0.05, ** showing *p* < 0.01).

QRTPCR was used to profile the expression of two sets of relevant genes, in both adults and in larvae, the life-cycle stage when the growth defect manifests in *tko*^*25t*^ [6]. We first analyzed a set of Malpighian tubule transporters proposed to be involved in the excretion of excess sugar [17]. Their expression was consistently elevated in *tko*^*25t*^, both in L3 larvae (Fig 2A) and adults (Panel A in S2 Fig), typically 3–4 fold. The most prominently expressed of these mRNAs, CG7882 and CG3285, were also tested for responsiveness to dietary sugar at larval stage. In both genotypes, their expression was elevated 2–3 fold on high-sugar compared with zero-sugar food (Fig 2C). Next we analyzed a set of α-glucosidases specific to, or highly enriched in the gut, and putatively involved in the mobilization of dietary sugars, using a similar approach. In both larvae (Fig 2B) and adults (Panel B in S2 Fig), these were down-regulated in *tko*^*25t*^. A prominently expressed representative of the set, CG9468, was further down-regulated by high-sugar diet in both genotypes (Fig 2D).

To understand the context of these changes in gene expression, we measured the total serum sugar concentration of *tko*^*25t*^ and control larvae grown on high-sugar versus zero-sugar media. *tko*^*25t*^ larvae had significantly lower levels of total serum sugars than wild-type larvae grown on the same medium. However, larvae cultured on high-sugar food had higher serum sugar levels than those of the same genotype grown on zero-sugar food (Fig 2E). *tko*^*25t*^ larvae also exhibited a lower rate of food consumption than control flies on the corresponding diet (Fig 2F), though on zero-sugar diet, where they grew faster, they paradoxically consumed less food than on high-sugar diet. In all these aspects, the phenotype of *tko*^*25t*^ larvae is consistent with a physiological strategy to minimize the amount of glucose, despite the initially presumed reliance on glycolysis.

### Metabolic derangement of fly larvae with mitochondrial dysfunction is exacerbated by high-sugar diet

**Fig 3:**
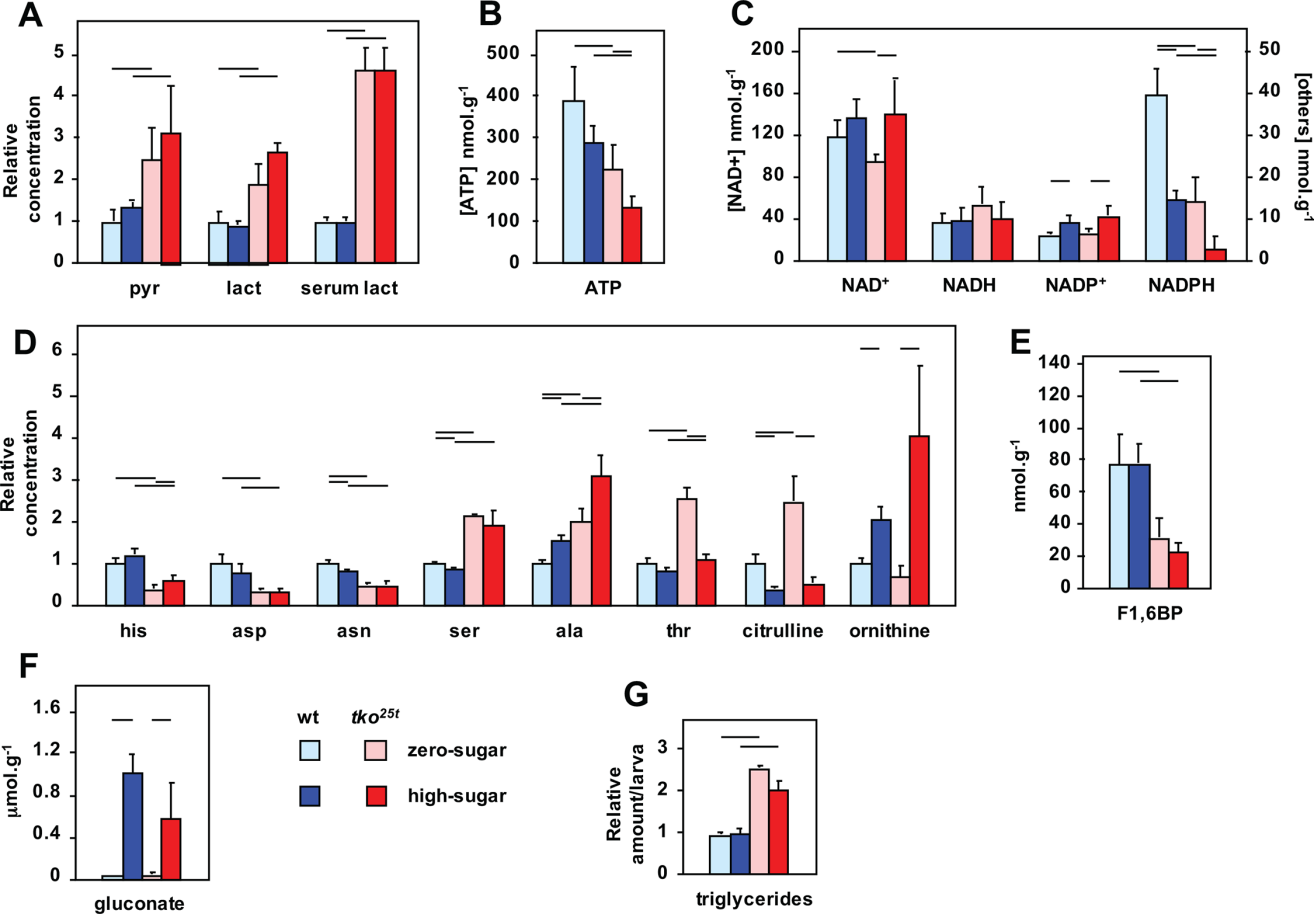
Metabolites showing substantial changes in *tko*^*25t*^ larvae. Relative levels of different metabolites in L3 larvae of the indicated genotypes and growth conditions, based on (A, B) findings from enzyme-linked assays or (C-F) mass spectrometry. Absolute values are shown for (B) ATP, (C) NAD+ and derivatives, (E) fructose 1,6-biphosphate (F1,6BP) and (F) gluconate, Values for (A) pyruvate, lactate and (D) amino acids are normalized to those for wild-type flies grown on ZS medium, enabling them to be plotted alongside for comparison. Relevant absolute values are given in S1 Table. Horizontal bars denote significantly different data classes (Student’s *t* test, *p* < 0.05). See S7 Table for fuller statistical analysis of metabolite levels. (G) Triglyceride levels in L3 larvae of the indicated genotype and growth conditions, normalized to the value for wild-type larvae grown on zero-sugar medium. Horizontal bars denote significantly different data classes (Student’s *t* test, *p* < 0.05). Note that we did not observe increased triglyceride levels when larvae were grown on high-sugar medium.

The previous findings of up-regulation of lactate dehydrogenase (LDH) expression in *tko*^*25t*^ adults [9] implied the use of LDH as an alternative pathway to regenerate NAD+, under conditions where mitochondrial respiration is limiting. We hypothesized that the resulting accumulation of lactate and/or the diversion of pyruvate from mitochondria may contribute to metabolic disturbance in *tko*^*25t*^, and underlie aspects of the mutant phenotype and its exacerbation by high-sugar diet. We therefore analyzed lactate and pyruvate levels in L3 larvae. The steady-state levels of both metabolites were found to be 2–3 fold elevated in *tko*^*25t*^ larvae compared with the wild-type control strain, when grown on either high- or zero-sugar medium (Fig 3A). When grown on high-sugar, this elevation was even more pronounced, at least for lactate, although the differences were at the border of significance. Serum lactate was also 4–5 fold elevated in *tko*^*25t*^ larvae (Fig 3A), and elevated lactate was maintained in adults (Panel A in S3 Fig).

Using mass-spectrometry (S1 Table) to gain a more complete insight into the metabolic abnormalities of *tko*^*25t*^ larvae, we observed a substantial ATP depletion (Fig 3B), as seen also in adults (Panel A in S3 Fig) [9]. ATP levels were decreased in high-sugar diet in both *tko*^*25t*^ and wild-type larvae, compounding the effects of genotype. NAD^+^ and NADH levels were only slightly altered by the *tko*^*25t*^ mutation or by diet (Fig 3C), but we observed a striking abnormality in the level of NADPH and the NADPH/NADP^+^ ratio (Fig 3C). In most physiological contexts, there is substantially more NADPH than NADP^+^, which was the case in control larvae grown on zero-sugar diet. The NADPH/NADP^+^ ratio was decreased both by high-sugar diet and by the presence of the *tko*^*25t*^ mutation. The effects of genotype and diet were again additive, so that in *tko*^*25t*^ larvae grown in high-sugar the ratio was reversed. In two of the four samples analyzed, NADPH was below the detection limit in mutant larvae grown on high sugar (S1 Table). *tko*^*25t*^ larvae also showed altered levels of some amino acids (Panel C in S3 Fig), notably a deficiency of histidine, aspartic acid and asparagine (Fig 3D), and elevated levels of serine, alanine (especially on high-sugar) and threonine (only on zero-sugar). Two other amino acid changes in *tko*^*25t*^ that differed between diets were of citrulline and ornithine (Fig 3D), amino acids implicated in growth regulation by virtue of their role in polyamine biosynthesis. Polyamines did show alterations in *tko*^*25t*^ (Panel D in S3 Fig), but the effect was the same, regardless of diet, whereas wild-type larvae showed clear increases in polyamine levels when grown on high sugar. Note that *Drosophila* has only an incomplete urea cycle, though urea was also greatly decreased in *tko*^*25t*^ larvae (S1 Table).

In addition to elevated pyruvate and lactate, we noted substantial alterations in the level of two other glucose metabolites: the glycolytic intermediate fructose 1,6-biphospate, threefold decreased in *tko*^*25t*^ larvae regardless of diet (Fig 3E), and the glucose oxidation end-product gluconate, >10-fold elevated on high-sugar diet irrespective of genotype (Fig 3F).

Finally, *tko*^*25t*^ larvae also showed a substantial triglyceride (TAG) accumulation not seen in control larvae (Fig 3G).

### Lactate and pyruvate accumulation contribute to growth retardation and NADPH depletion in larvae with mitochondrial dysfunction

**Fig 4:**
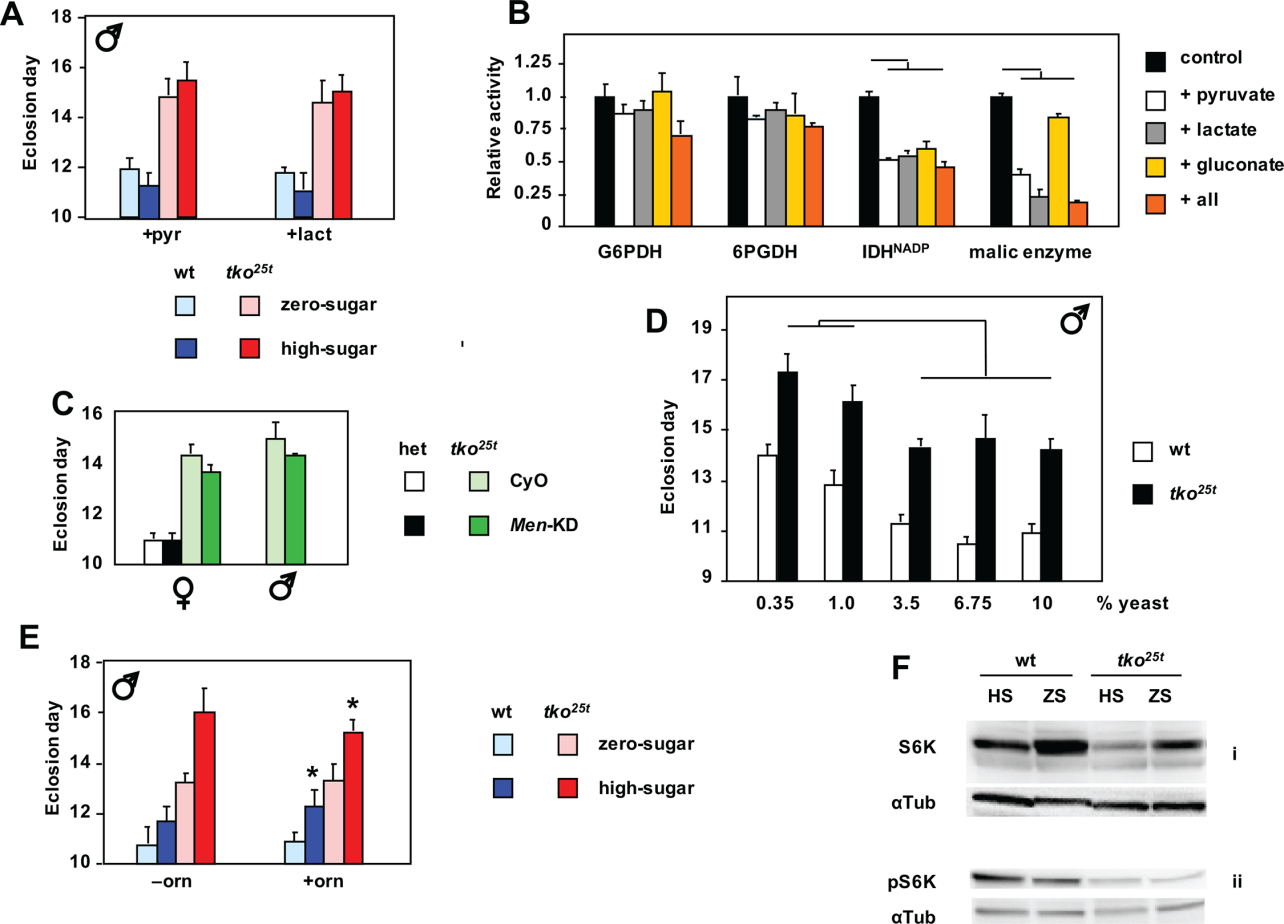
Metabolic phenotype of *tko*^*25t*^ and its modulation. (A) Time to eclosion of male flies of the indicated genotypes and growth conditions, on medium supplemented with pyruvate (pyr) or lactate (lact). In the presence of either supplement there were no significant difference in eclosion timing between *tko*^*25t*^ flies grown in high-sugar versus zero-sugar medium (Student’s *t* test, *p* > 0.05). See also Panel A in S4 Fig. (B) Effect on maximal activities of NADPH-producing enzymes from *Drosophila*, of the presence of excess amounts the indicated metabolites (all = pyruvate, lactate and gluconate together). For comparison, all such activities are normalized to those without any additions. Horizontal bars denote values significantly different from control (Student’s *t* test, *p* < 0.01). (C) Time to eclosion of flies of the indicated sex, genotype and growth conditions. het–flies heterozygous for *tko*^*25t*^ (which is recessive) and the FM7 balancer chromosome. Progeny carried either the CyO balancer chromosome marker or the RNAi construct for malic enzyme (*Men*-KD). (D) Time to eclosion of *tko*^*25t*^ and wild-type flies grown on media of the indicated composition (see S1 File for details). Horizontal lines indicate significant differences between data classes on different media, for a given genotype (Student’s *t* test, *p* < 0.05). On all media tested, eclosion times for *tko*^*25t*^ flies were also significantly different from those of wild-type flies grown on the same medium (Student’s *t* test, *p* < 0.01). For corresponding eclosion data of females see Panel B of S1 Fig. (E) Time to eclosion of male flies of the indicated genotypes and dietary conditions, on medium supplemented (or not) with ornithine (orn), as shown. * denotes value significantly different than for flies of the corresponding genotype and dietary condition, with ornithine *versus* to without the supplement (Student’s *t* test, *p* < 0.05). (F) Western blots of extracts from L3 larvae of the indicated genotypes and dietary conditions, probed for S6K or pS6K (phosphorylated at Thr-398) and the α-tubulin loading control (αTub). See also Panel F in S4 Fig.

We reasoned that the high levels of lactate and pyruvate seen in *tko*^*25t*^ larvae may limit flux through glycolysis, potentially accounting for a relative deficiency of ATP in animals largely dependent on glycolysis for ATP production. We therefore tested the effects of adding pyruvate or lactate to the culture medium. Lactate or pyruvate at 25 mg/ml, when added to zero-sugar food, increased the developmental delay of *tko*^*25t*^ flies by 1–2 days, partially phenocopying the effect of high-sugar diet (Fig 4A and Panel A in S4 Fig). The supplements also retarded the development of control flies. However, when added to high-sugar diet, they had only a minimal effect.

Next we considered whether the large changes in NADPH, which also correlated with the severity of the mutant phenotype on different diets, might also be influenced by excessive lactate and pyruvate. NADPH is required to drive biosynthetic reactions, notably fatty acid synthesis, but is also needed to maintain redox homeostasis. It is mainly produced by five ‘workhorse’ enzymes of catabolism (Panel B of S4 Fig), also linked to the provision of carbon skeletons for biosynthesis. We therefore measured the activity of these enzymes in extracts from *tko*^*25t*^ and control larvae, and the effects on their activities of the high levels of lactate and pyruvate, as well as gluconate. The maximal activities of the NADPH-producing enzymes were broadly similar in extracts from *tko*^*25t*^ and control larvae (Panel C in S4 Fig). However, when we added lactate or pyruvate at the high concentrations observed in *tko*^*25t*^ larvae grown in high sugar, we saw inhibition of three of the enzymes that contribute substantially to NADPH production (Fig 4B). The degree of inhibition of malic enzyme by pyruvate may be an under-estimate, since the *in vitro* assay is conducted in substrate excess, whereas *in vivo* the high levels of pyruvate should decrease throughput to very low levels, via product inhibition. Gluconate also had a minor effect on NADP-linked IDH activity. Taking account of the likely inhibition of malic enzyme *in vivo*, and assuming that the effects of the tested metabolites on the other enzymes are additive, this could partially explain NADPH depletion in *tko*^*25t*^ larvae.

Further down-regulation of malic enzyme by RNAi produced no significant worsening of the developmental delay, and might even have slightly alleviated it (Fig 4C), whilst effects on wild-type (*tko*^*25t*^ heterozygous) flies were minimal. A similar experiment to test for toxic effects of gluconate (or the failure to detoxify glucose), by down-regulating glucose dehydrogenase (Gld), could not be meaningfully executed, since global RNAi against Gld was developmentally lethal to both wild-type and *tko*^*25t*^ flies, as is the null mutant [18]. However, advanced glycation end-products did not accumulate in larvae grown on high-sugar diet (Panel B in S3 Fig), indicating that they cannot account for the deleterious effects of high-sugar diet on *tko*^*25t*^. However, as discussed below, the accumulation of gluconate in larvae grown on high-sugar medium implies a mechanism for the additional depletion of NADPH resulting from diet.

The disturbed amino acid levels in *tko*^*25t*^ larvae (Fig 3D) prompted us to consider whether a dietary deficiency or excess of amino acids might underlie aspects of the phenotype. To test this, we cultured flies on media containing a variable amount of yeast, the major source of dietary protein, but a fixed sucrose concentration at (7.5%). Varying the amount of yeast produced no change in the developmental delay of *tko*^*25t*^ flies (Fig 4D and Panel B in S1 Fig). Above a threshold level of 3.75%, yeast supplementation had no further effect on either *tko*^*25t*^ or control flies. At lower yeast concentrations, both were retarded further, but to the same extent, with males (Fig 4D) and females (Panel B in S1 Fig) affected similarly. Similarly, high-protein diet had no detrimental effect on flies grown on low-sugar (Fig 1C and Panels C-E in S1 Fig), and might even have had a slightly beneficial effect in the zero-sugar medium. Dietary supplementation with ornithine, found at unusually high levels in *tko*^*25t*^ larvae grown on high-sugar diet, had only very minor effects (Fig 4E and Panels D and E in S4 Fig).

### Growth inhibition in flies with mitochondrial dysfunction is associated with modulation of S6K

Since the developmental outcome in *tko*^*25t*^ is still a viable fly, its developmental delay must be the result of a coordinated signaling process with most likely both an intracellular and an endocrine component. In order to determine the molecular mechanism restraining growth in *tko*^*25t*^ larvae on different diets, we interrogated the status of major signaling pathways already known to be involved in growth regulation. The best characterized such pathway responding to intracellular energy status is that of AMPK [19]. ATP depletion against a constant level of AMP, as documented in *tko*^*25t*^ larvae, should lead to activation of AMPK by phosphorylation at Thr-172 (human numbering), which then down-regulates many downstream growth-related functions. Western blotting using AMPK-specific antibodies, revealed increased levels of phosphorylated AMPK in *tko*^*25t*^ compared to wild-type larvae (Panel F, sub-panels i and ii in S4 Fig) which was further enhanced on high-sugar diet, although none of these changes was dramatic. The best-characterized response pathway to extracellular signals, including diet and insulin/insulin-like growth factor signaling (IIS), involves the kinase Akt, whose activation by phosphorylation at serine-505 has a growth-promoting readout that also facilitates glucose-related metabolism [20]. Accordingly, growth of wild-type larvae on high-sugar diet led to a modest increase in the phosphorylated form of Akt (Panel F, sub-panels iii and iv in S4 Fig,) which, taking account of the loading control, appeared to be partially abrogated in *tko*^*25t*^. The Akt and AMPK pathways converge on mTOR, the key regulator of cytosolic protein synthesis and growth, whose canonical readout is the phosphoryation status of S6K [21]. Consistent with this, we observed a decreased amount of the isoform of S6K phosphorylated at Thr-398 (one of the mTOR target sites) in *tko*^*25t*^ compared with wild-type larvae, although in both genotypes the level of this isoform appeared marginally higher on high-sugar than zero-sugar diet (Fig 4F, panels ii). More strikingly, we observed a substantial decrease in total S6K in *tko*^*25t*^ larvae, specifically on high-sugar diet (Fig 4F, panels i; biological replicates in Panel F, sub-panels v and vi in S4 Fig). Analysis, by QRTPCR, of mRNA levels of the major insulin-like peptides expressed in larvae, showed a consistent, but quantitatively minor up-regulation in *tko*^*25t*^ (Panel G in S4 Fig; see also S2 Table).

### Cystolic protein synthesis and secretion are key targets of the metabolic crisis caused by mitochondrial dysfunction on high-sugar diet

Analysis of gene expression at the RNA level by global RNA sequencing revealed systematic and diet-dependent changes in *tko*^*25t*^ larvae that can be construed as a combined readout from metabolic disturbance and decreased growth-signaling. We analyzed the data in two ways: firstly, according to the largest proportionate changes, and secondly, the largest absolute changes, (S3 Table and S4 Table, respectively). We also analyzed separately the changes in protein-coding genes (Sheets A and C of S3 Table and Sheets A and C of S4 Table) and non-coding RNAs (Sheets B and D of S3 Table and Sheets B and D of S4 Table), and executed all comparisons both by genotype (Sheets A and B of S3 Table and Sheets A and B of S4 Table) and by diet (Sheets C and D of S3 Table and Sheets C and D of S4 Table: see also the full, unselected data in S2 Table). The following summary conclusions emerge.

As regards the non-coding RNAs, despite some striking changes in abundance, very few of them are functionally assigned at this time, and thus cannot be assessed further. The largest absolute changes in protein-coding genes were seen mostly in those which were already highly expressed, and fell into well-defined functional classes. Wild-type larvae on high-sugar diet, compared with those grown on zero-sugar diet (Sheet C in S3 Table), showed strong induction of genes connected with cytosolic translation, glycolysis, the structural proteins of muscle and the larval cuticle, whist the most strikingly down-regulated genes were gut-specific, and connected with digestive functions, including proteases, lipases, lysozymes, components of the peritrophic membrane and other chitin-binding proteins. In contrast, the vast majority of these genes were either less responsive, unaltered or even oppositely regulated by high-sugar diet in *tko*^*25t*^ larvae, where digestive functions were amongst the most prominently up-regulated genes. Comparing *tko*^*25t*^ with wild-type larvae (Sheet A in S3 Table), mitochondrial transcripts were down-regulated on both diets, whilst the most striking differences on high-sugar diet were those already indicated above. Intriguingly, whilst many cytosolic translational components were down-regulated in *tko*^*25t*^ on high sugar, one that was up-regulated was the inhibitory factor Thor (4E-BP). Many of the genes showing the largest proportionate changes remain functionally unassigned, but genotype-specific changes were rather similar on the two diets (Sheet A in S3 Table), whereas diet-specific changes (Sheet C in S3 Table) were largely different between the two genotypes. One of the most highly induced genes in *tko*^*25t*^ larvae irrespective of diet was p24-2, whose mRNA was induced over 1000-fold compared with its level in control larvae. p24-2 is one of two fly homologues of yeast p24, a protein required for vesicle trafficking in the secretory pathway. Up-regulation of p24-2 suggests ER stress, but we saw no changes in the level or splicing pattern of Xbp1 RNA (Panels H and I in S4 Fig), considered a marker for the induction of the classic ER stress response. Other prominent changes in *tko*^*25t*^ larvae involved genes involved in antimicrobial defense. An unbiased annotation enrichment analysis of all genes showing at least two-fold changes in expression (S5 Table), executed using the DAVID (Database for Annotation, Visualization and Integrated Discovery, http://david.abcc.ncifcrf.gov) gene ontology database tools, reached broadly similar conclusions.

### *tko*^*25t*^ is immune to the growth-inhibitory effects of cytosolic protein synthesis inhibition

The strikingly opposite sugar-dependent regulation of highly expressed genes for the machinery of cytosolic protein synthesis and secretion in *tko*^*25t*^ compared with wild-type larvae could, in principle, be construed as the cause or consequence of the metabolic disturbances and growth impairment: in other words it could be an adaptive response to limit growth under deranged metabolic conditions, or could be a maladaptive consequence of such disturbances. Thus, we reasoned that imposing an additional stress on the protein synthetic apparatus would worsen the phenotype if the above changes are maladaptive, but would be neutral or possibly even beneficial, if down-regulation of protein synthesis and secretion were part of an adaptive response.

**Fig 5:**
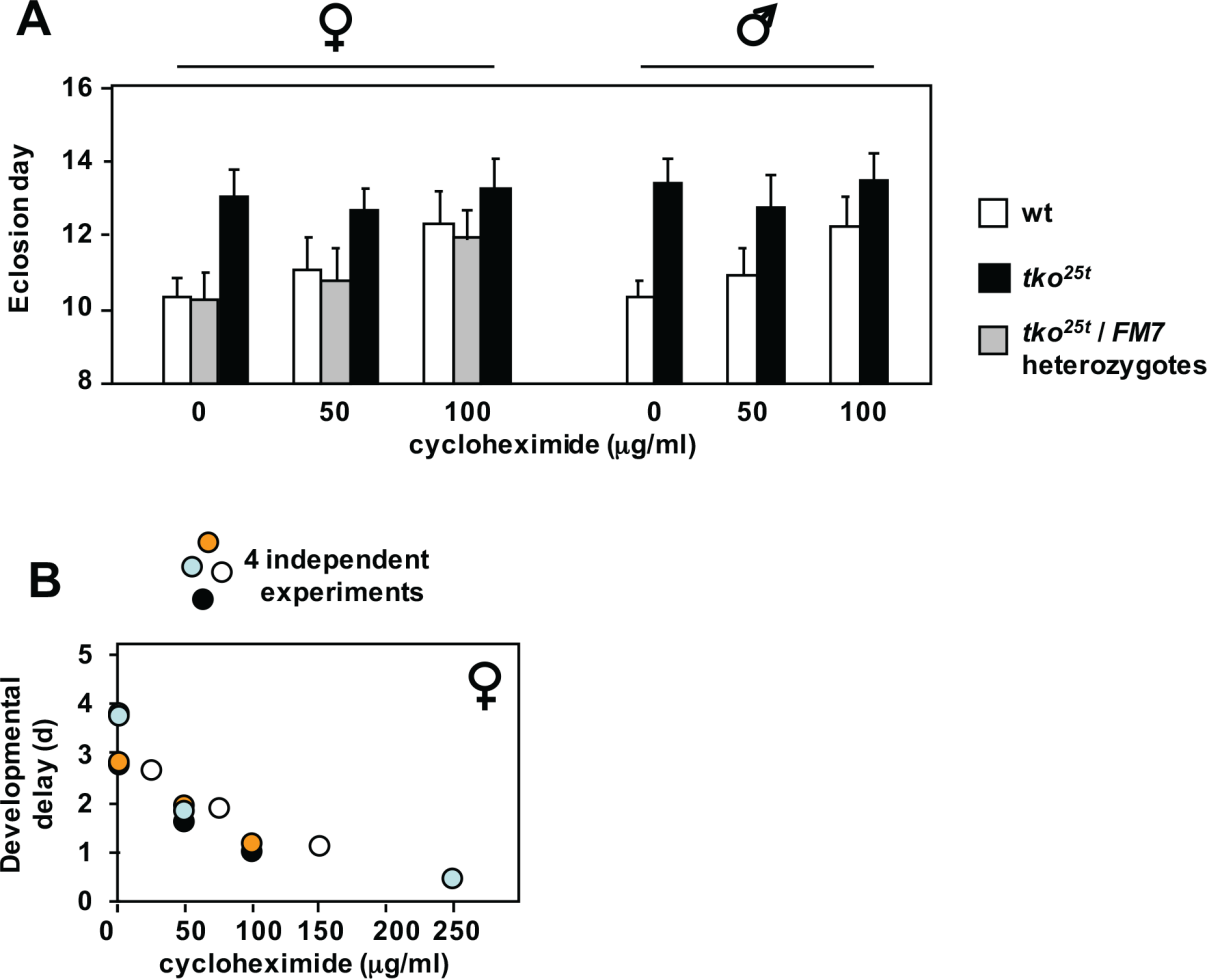
Effect of cycloheximide on development of *tko*^*25t*^ and wild-type flies. (A) Means ± SD of times to eclosion of flies of the sex and genotypes indicated, on high-sugar medium, with or without cycloheximide at the indicated concentrations. Based on pairwise *t* tests, and considering all the flies of a given sex and genotype cultured at a specific drug concentration as a single population, mean eclosion times were significantly different (*p* < 0.05) at different cycloheximide concentrations, apart from *tko*^*25t*^ flies of either sex at 100 μg/ml, which were not different from *tko*^*25t*^ flies cultured in the complete absence of the drug. (B) Pooled eclosion data from four independent experiments conducted with different concentration ranges of cycloheximide. Female developmental delay showed consistent decrease with increasing cycloheximide concentration. Males showed same trend (Panel C in S5 Fig).

To address this, we cultured wild-type and *tko*^*25t*^ flies on media containing low doses of cycloheximide, an inhibitor of cytosolic translational elongation. Cycloheximide produced a dose-dependent growth retardation in wild-type flies, but had almost no effect on *tko*^*25t*^ mutant flies, if anything tending to accelerate their development very slightly, especially when analyzing the progeny only from eggs laid on the first day of exposure to the drug, thus minimizing any confounding effects on egg-laying behaviour or oogenesis (Fig 5A and 5B and Panels A-C in S5 Fig). We also noted a tendency toward alleviation of bang-sensitivity (Panel D in S5 Fig), although this was not statistically significant due to the large variances in recovery time. In addition, *tko*^*25t*^ flies were immune to the growth-retarding effects of tunicamycin seen in wild-type flies, when the drug was added to the growth medium at the highest practical dose of 12 μM (Panel E in S5 Fig).

### The gut is a crucial tissue for the developmental delay phenotype of *tko*^*25t*^ flies

**Fig 6:**
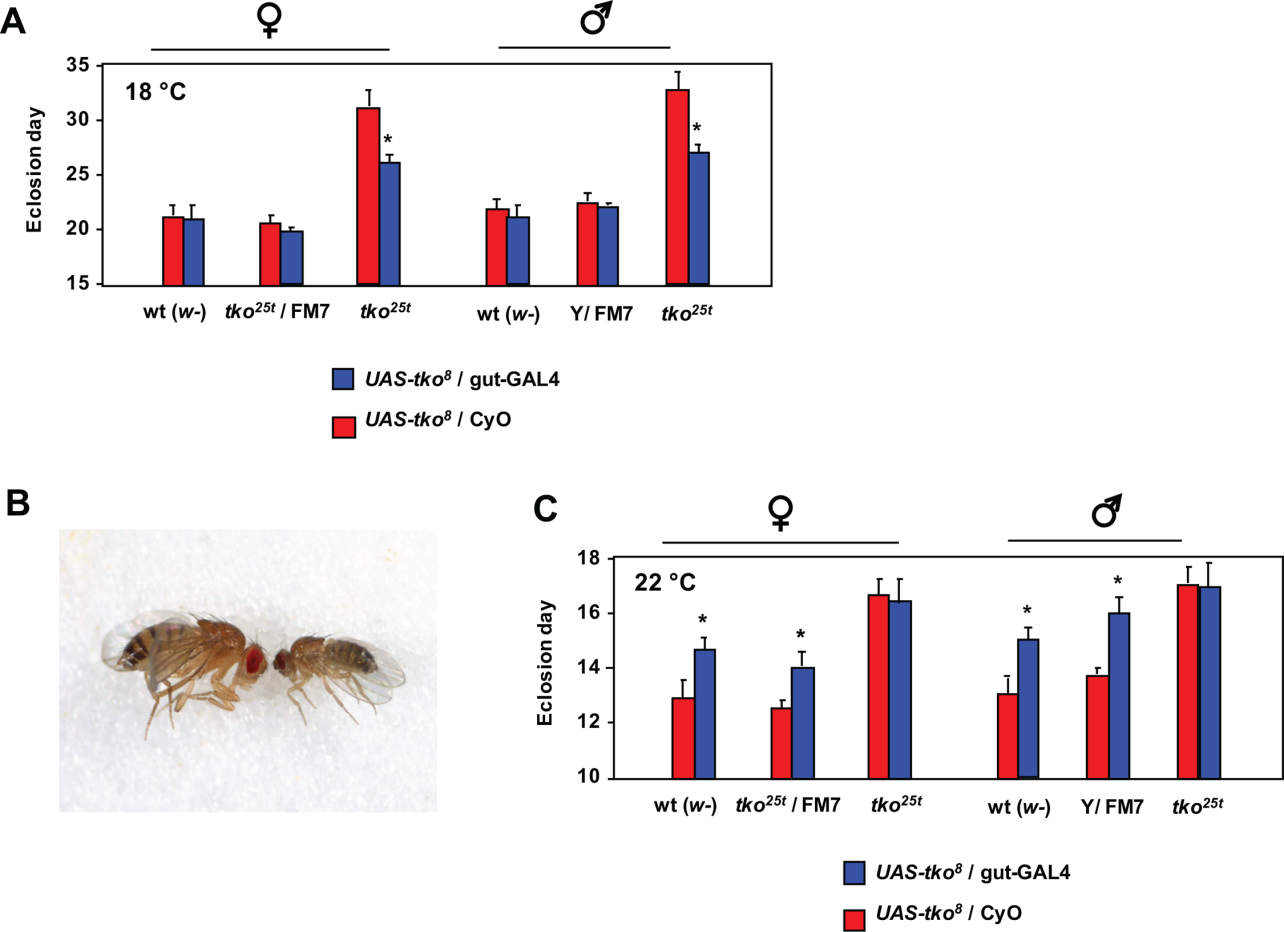
Partial rescue of *tko*^*25t*^ by gut-specific expression of *tko*. (A, C) Means ± SD of times to eclosion of flies of the sex and genotypes indicated, on high-sugar medium, at (A) 18°C or (C) 22°C. Asterisks denote significant differences in pairwise *t* tests (*p* < 0.01) between flies of a given sex and genotype carrying the gut-GAL4 driver (Kyoto line 113094) compared with the corresponding flies carrying the CyO balancer chromosome instead. (B) Minute phenotype of the few flies eclosing with the genotype *UAS*-*tko*^*8*^ / gut-GAL4 when cultured at 26°C.

The striking down-regulation of components of the machinery of cytosolic protein synthesis and secretion in *tko*^*25t*^ larvae, combined with the observation that the effect of the mutation is epistatic to the developmental delay introduced by low doses of cycloheximide, suggests that one or more tissues responsible for the synthesis of secreted proteins are key targets of the mutation. An obvious candidate is the gut, especially since many secreted gut proteins showed oppositely altered expression in *tko*^*25t*^ compared with wild-type larvae. We therefore tested whether gut-specific expression of the wild-type *tko* gene in the *tko*^*25t*^ background could alleviate the mutant phenotype. To do this, we made use of a previously constructed transgenic line containing a GAL4-dependent copy of *tko* (*UAS*-*tko*^*8*^) [22], which we combined with a gut-specific GAL4 driver line (Kyoto 113094, S6 Fig), in the *tko*^*25t*^ and wild-type backgrounds. The effect was extremely temperature-dependent. At 18°C the *UAS*-*tko*^*8*^ and Kyoto 113094 driver combination had no significant effect in the wild-type background, but *tko*^*25t*^ flies showed a clear, though partial rescue of their very long developmental delay at this temperature (Fig 6A). At 26°C the combination of *UAS*-*tko*^*8*^ and the Kyoto 113094 driver was lethal or semi-lethal, with just a small number of escaper flies eclosing in the wild-type background, with an ~4 day developmental delay, and exhibiting a minute phenotype (Fig 6B). At 22°C the combination remained deleterious in the wild-type background, with flies showing a 1–2 day developmental delay, but in the *tko*^*25t*^ background there was no such effect (Fig 6C).

## Discussion

Our starting point in this study was the erroneous assumption that the limited capacity of OXPHOS in *tko*^*25t*^ flies would render them highly dependent on glycolysis, and thus addicted to sugar. However, increasing the sugar content of their diet instead led to exacerbation of the mutant phenotype. Comparisons of larvae grown on different diets revealed a set of metabolic disturbances in *tko*^*25t*^ larvae (high pyruvate and lactate, low ATP and NADPH) that were generally accentuated by high-sugar diet. Growth in high-sugar produced strikingly opposite changes in gene expression in wild-type and mutant larvae, notably affecting the apparatus of cytosolic protein synthesis and secretion. In addition, we observed altered cell signaling, converging on the cytosolic translation machinery, whilst *tko*^*25t*^ was immune to the growth inhibitory effects of low levels of cycloheximide, an inhibitor of cytosolic translation.

### Key metabolic consequences of mitochondrial dysfunction

Many of the metabolic disturbances seen in *tko*^*25t*^ larvae are consistent with previous studies of OXPHOS deficiency, although their exacerbation by high-sugar diet is novel. Elevated lactate, pyruvate and alanine are common findings in mitochondrial disease patients [23], considered to represent increased dependence on glycolysis under conditions where mitochondrial NADH re-oxidation is impaired [24]. *tko*^*25t*^ larvae showed decreased levels of the glycolytic intermediate immediately upstream of the rate-limiting step, i.e. fructose 1,6-biphosphate (Fig 3E), consistent with increased glycolytic flux. Lactate accumulation may reflect the need for lactate dehydrogenase as an alternative pathway to regenerate NAD+ but, since it is accompanied by high pyruvate, may also be a consequence of decreased TCA cycle capacity due to the OXPHOS defect. Pyruvate accumulation may be partially adaptive, by feeding anaplerotic pathways, but addition of dietary pyruvate (or lactate) to *tko*^*25t*^ partially phenocopied the effects of high-sugar diet, implying these end-products of glycolysis to be important agents of the metabolic crisis that limits growth, even if only via product inhibition of glycolytic flux, thus compromising the maintenance of ATP levels. The reason for the additional burden of pyruvate and lactate resulting from high-sugar diet is not immediately obvious. It may partly be a consequence of increased substrate availability for glycolysis and other catabolic pathways that converge on pyruvate. Logically, however, it also reflects maladaptive effects of sugar-dependent signaling.

Whist *tko*^*25t*^ larvae are able to maintain normal NAD+/NADH levels, NADP+/NADPH homeostasis is severely disturbed (Fig 3C). As well as its role as an electron donor for biosynthesis, NADPH buffers oxidative stress, most importantly as a cofactor in the regeneration of the reduced form of thioredoxin [25], which in *Drosophila* replaces the functions of glutathione reductase [26]. Mitochondria from *tko*^*25t*^ flies produce excess ROS [16], potentially accounting in part for NADPH depletion. High levels of pyruvate and lactate, which we demonstrate to inhibit key NADPH-generating enzymes (Fig 4B), notably IDH and malic enzyme, may also play an influence.

The further depletion of NADPH in *tko*^*25t*^ grown in high sugar (Fig 3C) appears to be a compounding of these effects with an independent effect of high-sugar diet. Excess unmodified glucose is toxic because of its ability to react with the lysine side-chains of proteins, bringing about their irreversible inactivation via the Maillard reaction [27, 28]. This toxicity is believed to play a major role in the pathology of diabetes (reviewed in [29]), and organisms use a variety of strategies to minimize exposure of the tissues to excess glucose. *Drosophila* can detoxify glucose via the FAD-linked enzyme glucose dehydrogenase (Gld, CG1152, EC 1.1.99.10), which produces gluconate as an inert end-product, avoiding the generation of peroxide [30, 31]. Thus, as predicted, gluconate accumulates in larvae grown on high-sugar medium (Fig 3F). Although the exact electron acceptor for Gld is not known [31], its regeneration should depend on the cytochrome P450 system, in which terminal oxidation is coupled to NADPH consumption, in the general reaction A-H2 + 2NADPH + O2 → 2H2O + 2NADP+ A (where A is a generic electron acceptor). An effect on NADPH is evident even in wild-type larvae (Fig 3C), although it does not appear to impair development in the absence of the other metabolic disturbances seen in *tko*^*25t*^. However, its compounding with the effects of OXPHOS deficiency in *tko*^*25t*^ leads to the highly abnormal situation of a reversal in the NADPH/NADP+ ratio that must have serious consequences for many cellular processes. Notably, the processing of secreted proteins in the ER is highly dependent on NADP+/NADPH homeostasis [32, 33]. Disturbed proteostasis in the secretory pathway [34] would be predicted to affect those tissues most exposed to dietary glucose and most dependent on secretion, i.e. the gut and its associated organs, and the epidermis, which secretes the larval cuticle at each molt. This is concordant with the altered patterns of gene expression that we observed (S4 Table).

The ability of the *Drosophila* larval gut to mount a classic ER stress-response is limited. Xbp1, the key transcription factor required to effect the response, is already reported to be constitutively activated in the larval gut [35], consistent with our own findings (Panerls H and I of S4 Fig). Neither diet nor the *tko*^*25t*^ mutation had any further effect on its global expression level or splicing, suggesting that there is only a limited capacity for handling further proteotoxic stress in the ER. The activation of accessory pathways for secretion, such as involving p24-2 (Sheet A in S3 Table), may be a signature of this. P24 proteins are involved in the sorting of glycosylphosphatidylinositol (GPI) anchored proteins into COPII vesicles [36, 37] which then transit to the cell surface via the Golgi. The down-regulation, at the RNA level, of the apparatus of cytosolic protein synthesis and secretion (Sheets A and C in S4 Table), combined with active mechanisms to attenuate both processes (Fig 5 and S5 Fig), such as via S6K modulation (Fig 4F and Panel F in S4 Fig), can be rationalized as a response to proteotoxicity in the ER, due to redox disturbance.

ATP deficiency is a frequently observed or predicted effect of OXPHOS insufficiency, including previous reports on *tko*^*25t*^ and other *Drosophila* mutants [10, 12, 16, 38, 39]. However, decreased OXPHOS capacity and limitations on glycolysis due to high pyruvate and/or lactate may not be the only reasons for ATP depletion. Protein folding and refolding are highly ATP-consuming processes, as is the proteolytic degradation of misfolded or aggregated proteins [40]. Disturbed ER proteostasis in the ER may therefore be a drain on already decreased levels of ATP.

The consequences of the amino acid abnormalities detected in *tko*^*25t*^ larvae are less clear. Deficiencies in specific amino acids are expected to trigger growth arrest and inhibition of cytosolic protein synthesis through the TOR pathway independently of the effects of ATP depletion. However, dietary supplementation with protein did not rescue *tko*^*25t*^. The amino acid imbalances in mutant larvae (Fig 4D) are therefore best considered as a side-effect of pyruvate overload that, on their own, are insufficient to trigger growth arrest. Excess alanine, also seen in patients with elevated pyruvate [23, 41], probably results from the action of alanine aminotransferase [42]. Similar arguments may apply to serine. The abnormalities in ornithine and citrulline (Fig 3D), combined with the observed changes in mRNAs for enzymes involved in their metabolism (S3 and S4 Tables), are harder to interpret, as are effects on polyamine levels, none of which offer any explanation for growth limitation in *tko*^*25t*^ (Panel C in S3 Fig). Moreover, ornithine supplementation in zero-sugar diet did not phenocopy the effects of high sugar, and may even have slightly alleviated it (Fig 4E).

**Fig 7:**
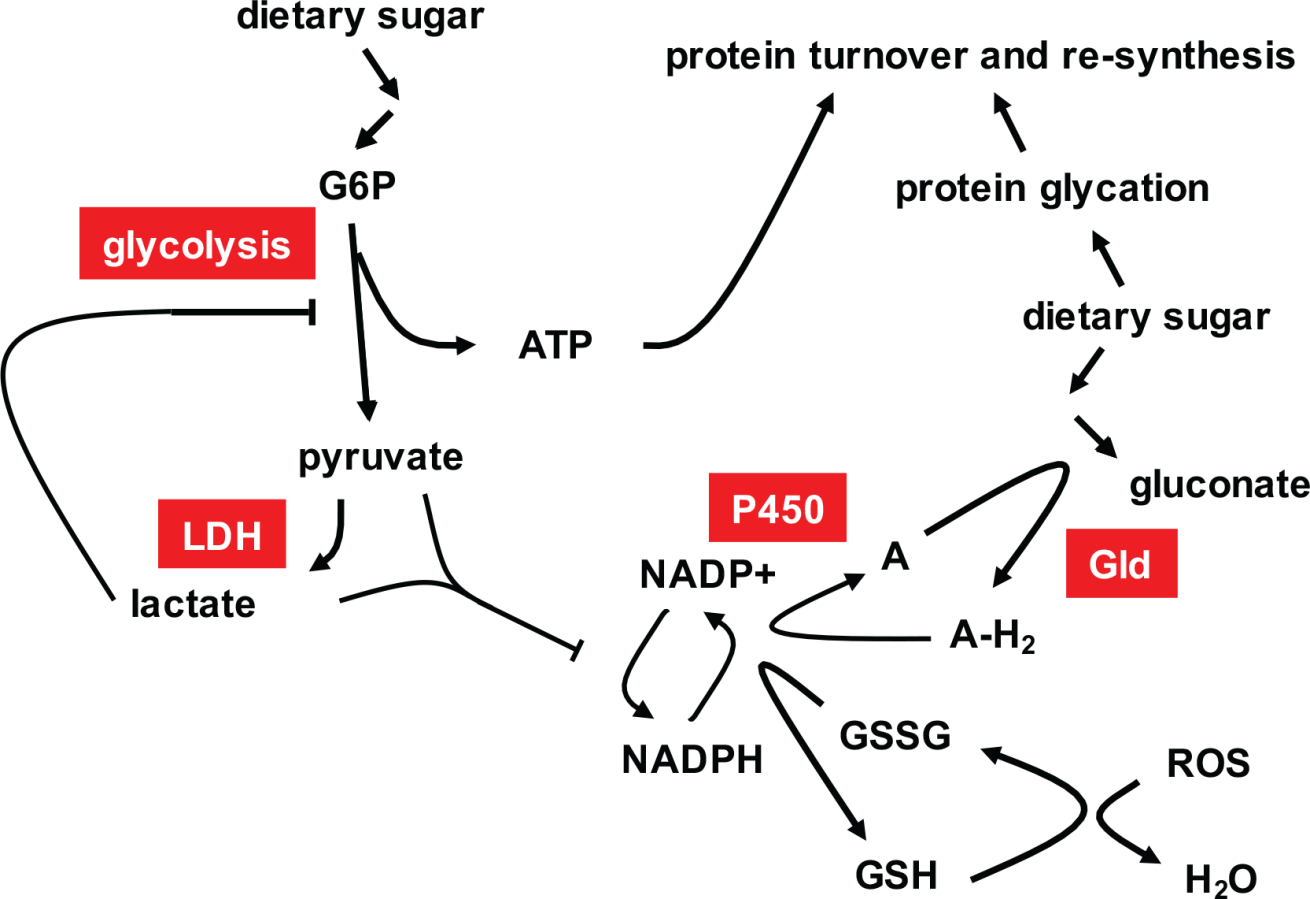
Summary model of the metabolic phenotype of *tko*^*25t*^ in high-sugar medium. High dietary sugar and mitochondrial dysfunction stimulate glycolysis, which is nevertheless limited by the build up of pyruvate and/or lactate, produced by lactate dehydrogenase (LDH, needed to regenerate NAD+ when OXPHOS is impaired), restricting ATP production. Pyruvate and lactate accumulation restricts NADPH production, whilst NADPH consumption is increased by the need to maintain glutathione in the reduced state, despite increased mitochondrial ROS production, and by the need to reoxidize the electron acceptor (A) for glucose dehydrogenase (Gld) using the P450 system, preventing further peroxide generation. Glucose dehydrogenase converts excess glucose to gluconate, whilst any glycotoxic damage is compensated by the normal processes of protein refolding, turnover and re-synthesis, which consume ATP.

Since it is difficult to manipulate the levels of pyruvate, ATP and NADPH independently, their relative contributions to the developmental phenotype are difficult to apportion. Knockdown of *Men* (malic enzyme), for example, produced no exacerbation and possibly a slight alleviation of the phenotype (Fig 4C). However, any decrease in flux should directly decrease both NADPH and pyruvate, whilst decreased pyruvate may in turn allow an increased rate of NADPH production and facilitate glycolytic flux. Unknown allosteric effects, undetected metabolites, subcellular compartmentalization of substrates, products and enzymes, and tissue-specificity of metabolic pathways may impact the diet-dependent metabolic crisis of *tko*^*25t*^, summarized in Fig 7.

### Growth-related signaling

The metabolic crisis of *tko*^*25t*^ does not lead to lethality or grossly abnormal development, but to a decreased developmental rate, tailoring growth to resources and the ability to mobilize them, and resulting in a viable adult fly. This indicates the operation of a programmed response mechanism. We postulate that, in the wild, this would allow the fly to survive in different nutritional environments, perhaps including those in which it may be exposed to toxins targeted on OXPHOS or mitochondrial translation. The response clearly has both a transcriptional and post-translational dimension, though the underlying signaling machineries may overlap, and may also operate tissue-specifically, accounting for the many changes in expression of genes expressed uniquely in the gut, muscle or epidermis. Furthermore, although transcriptional responses most likely underlie the major changes in mRNA levels, we cannot exclude a contribution from non-coding RNAs regulated in response to the *tko*^*25t*^ mutation or the sugar content of the diet.

We found evidence for the involvement of several well-characterized signaling pathways converging on cytosolic protein synthesis, notably including the ATP-responsive AMPK and nutrient-sensitive (Akt) pathways, as well as the key target of TOR signaling, S6K (Fig 6B). Strikingly, *tko*^*25t*^ flies were immune to the growth-inhibitory effect of low doses of cycloheximide, consistent with the translational apparatus being a key downstream target of regulation in the mutant. This is unlikely to be due to drug exclusion. Although induced in *tko*^*25t*^ adults [9], the Mdr-related transporter l(2)03659 is expressed at slightly lower levels in *tko*^*25t*^ than wild-type larvae (S2 Table) and is not sugar-responsive. Moreover, *tko*^*25t*^ flies are more sensitive than wild-type flies to doxycycline [6], an inhibitor of mitochondrial rather than cytosolic protein synthesis.

Cycloheximide inhibits the elongation step of the ribosome cycle [43], which is normally not rate-limiting. Instead, initiation is the target of most translation-regulatory mechanisms, e.g. via the protein kinases targeting initiation factors such as eIF-2A or eIF-4E [44]. However, it would appear that initiation is already working at close to maximal capacity in wild-type *Drosophila* larvae, since cycloheximide inhibits growth in a dose-dependent manner even at low concentrations. In contrast, at doses up to 100 μg/ml, *tko*^*25t*^ flies were refractory to cycloheximide (Fig 5B and Panel C in S5 Fig), indicating a condition where initiation, rather than elongation, is constrained, and is thus rate-limiting for protein synthesis and growth. The altered levels of phosphorylated isoforms of known signaling molecules is consistent with the implied down-regulation of translational initiation in *tko*^*25t*^, and its exacerbation by high-sugar diet. Thus, while total levels of the AMPK α-subunit are little altered, *tko*^*25t*^ flies show increased amounts of the isoform phosphorylated at Thr-172, which attenuates TOR signaling [19], but a corresponding decrease in the isoform of Akt phophorylated at Ser-505, which activates TOR. The increase in AMPK phosphorylation is consistent with the observed ATP depletion [45], but the mechanism by which Akt signaling attenuated remains unknown, and could involve any of the metabolic changes associated with mitochondrial dysfunction.

Consistent with these changes, *tko*^*25t*^ shows decreased amounts of the isoform of the key TOR target [46] S6K, phosphorylated at Thr-398, which in turn activates translational initiation [47]. However, all of these changes are rather modest, raising doubts as to whether they, alone, could account for an effective halving of the growth rate during larval development.

**Fig 8:**
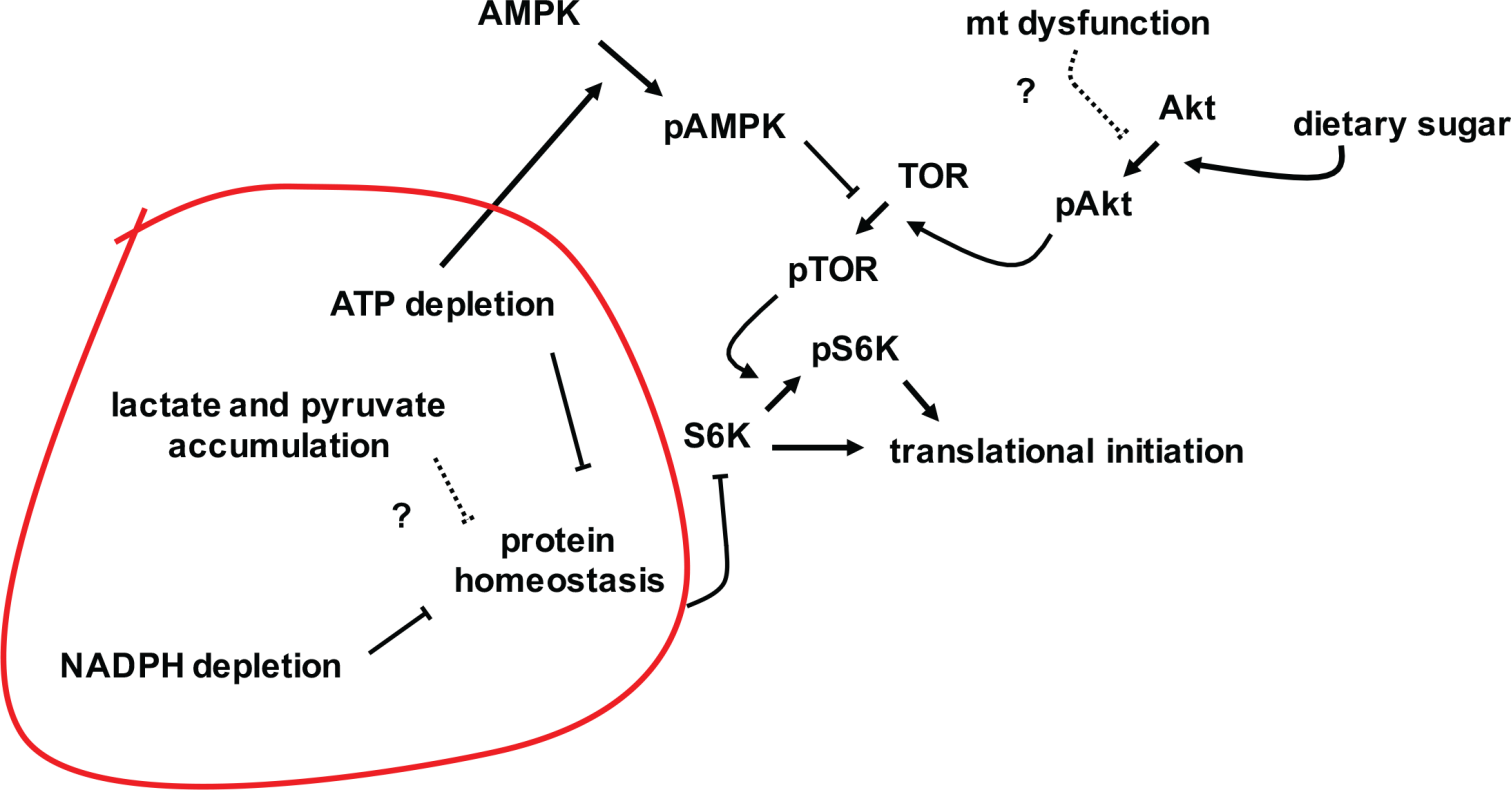
Summary model of the growth phenotype of *tko*^*25t*^ in high-sugar medium. NADPH depletion disturbs protein homeostasis in the ER (and may also be exacerbated by it, in a vicious cycle) consuming further ATP. ATP depletion activates AMPK, inhibiting TOR complex activation by phosphorylation (denoted as pTOR), and consequently limiting the activation of S6K and other growth-stimulatory effectors by phosphorylation. Mitochondrial dysfunction is proposed to inhibit Akt activation by an unknown process (dotted line), limiting its activation by dietary sugar. One or more of lactate/pyruvate accumulation, ATP and NADPH depletion, or their combined effects on protein homeostasis (red line), are proposed to stimulate S6K turnover, thus contributing further to the down-regulation of protein synthesis.

The most important new link between S6K and growth regulation is our finding that the level of S6K protein itself (specifically, the homologue of human S6K1) is strongly decreased in *tko*^*25t*^ larvae grown on high-sugar medium (Fig 4F and Panel F in S4 Fig). A recent report of the effects on *Drosophila* lifespan of different diets, combined with knockdown of an ATP synthase subunit [48], also showed modulation of the levels of S6K protein. Although less dramatic than the S6K changes observed here, this may indicate a parallel or accessory stress-response pathway, linking diet, mitochondrial function and regulation of cytosolic translation. Neither Sun et al. [48] nor ourselves were able to address the tissue specificity of S6K modulation, and the underlying molecular mechanism remains unknown, but our data raise a number of interesting, non-mutually excusive possibilities. The first is that further activation of AMPK in susceptible tissues (Panel F in S4 Fig), resulting from the more profound depletion of ATP (Fig 3B), triggers S6K turnover, e.g. via the ubiquitin-proteasome system. A second would invoke a regulator akin to AMPK, but activated instead by the abnormal NADPH/NADP+ ratio. A third such putative regulator would be sensitive to elevated gluconate (Fig 3F), which may be considered a specific indicator of the stress of high dietary sugar in the fly. A further possibility is that S6K levels are regulated by the downstream consequences of these metabolic changes, for example in response to proteotoxic stress or translational imbalances (see summary model, Fig 8).

The functional differences between modified and unmodified isoforms of S6K are poorly understood, but are likely to extend beyond global regulation of translation [49]. Many of the large quantitative differences in gene expression at the RNA level seen in *tko*^*25t*^ grown in high sugar were opposite to those induced by high-sugar diet in wild-type flies, notably those affecting the machineries of cytosolic protein synthesis and secretion as well as glycolysis. This suggests a regulatory event targeting the system responsible for glucose induction of gene expression which includes transcription factors such as sugarbabe [50] and Mondo/Mlx [51], but may also have a post/transcriptional component. These factors, as well as any proteins that interact with them directly or indirectly, should thus be considered as candidates for regulation by S6K.

We did not identify a specific systemic response mechanism to mitochondrial dysfunction in *tko*^*25t*^ that would correspond with the endocrine biomarkers GDF15 [52] or FGF21 in mammals [53]. Neither GDF15 nor FGF21 have orthologues in *Drosophila*, although both are members of well-characterized growth factor families that are well represented in the fly. No members of these families showed significantly altered expression at the RNA level in *tko*^*25t*^, although this does not preclude regulation at other levels. Expression of insulin-like peptides was also slightly increased, not depressed in *tko*^*25t*^ (Panel G in S4 Fig). Whilst the down-regulation of growth in *tko*^*25t*^ might be at least partially cell-autonomous, the fact that it appears to emanate from a deficiency of mitochondrial protein synthesis in a specific tissue (i.e. the gut, Fig 6), and that one readout is altered feeding behaviour (Fig 2D), it is likely to involve either a known [54] or still unknown endocrine circuit centered on the gut.

### Tissue-specificity of the effects of mitochondrial dysfunction in development

Five pieces of evidence implicate the gut as a crucial tissue mediating the growth retardation of *tko*^*25t*^ larvae, particularly its enhancement in high-sugar diet. First, based on dye ingestion, we observed a decreased food intake (Fig 2F). Second, specific gut alpha-glucosidases were down-regulated (Fig 2A), and further down-regulated in high sugar (Fig 2D), suggestive of decreased sugar absorption and accounting, at least partially, for decreased serum sugar (Fig 2E). Third, mRNAs coding for gut-specific enzymes were amongst those most responsive to diet and genotype, many showing clearly opposite behaviour in *tko*^*25t*^ compared with wild-type (Sheets A and C in S4 Table). The transcriptional readout of mitochondrial dysfunction in relation to diet is therefore largely gut-specific. Of note also is the fact that, in high sugar, *tko*^*25t*^ larvae showed down-regulation of many mRNAs coding for components of the machineries of cytosolic translation and secretion (Sheet A in S4 Table) and, at the protein level, of S6K (Fig 4F and Panel F in S4 Fig), considered a key regulator of translation, whilst the gut is one of the most important secretory organs. Fourth, the additional metabolic stress arising from NADPH depletion (Fig 3C) as a by-product of glucose oxidation must logically occur in a tissue exposed to high sugar levels. Given the fact that serum sugar is not elevated in *tko*^*25t*^ (Fig 2E) the only major tissues exposed to high sugar should be the epidermis and the gut. Finally, when directly tested using a gut-specific driver at 18°C, expression of the wild-type *tko* gene was able partially to rescue the developmental delay of *tko*^*25t*^ (Fig 6A), implicating the gut as a major tissue where insufficiency of mitochondrial protein synthesis impairs growth. The effect of GAL4 drivers is known to be temperature-dependent [55], with increased activity at 25°C compared with 18°C. Intriguingly, gut-specific over-expression of wild-type *tko* in the wild-type but not the *tko*^*25t*^ background at 22°C also produced a developmental delay (Fig 6C), suggesting that the expression level of the *tko* gene product (mitoribosomal protein S12), which in bacteria is a key component of the small subunit assembly pathway [56], may need to be tightly regulated in order to ensure the appropriate ratio of mitochondrial and cytosolic translational capacity. Over-expression of *tko* at even higher temperatures resulted in a phenotype of semi-lethality, with escapers having a classic minute phenotype (Fig 6B), seen previously in mutants with cytoribosome deficiency [57] or defective insulin signaling [58], but not in *tko*^*25t*^. The finding further highlights the importance of coordinated protein synthesis in cytosolic and mitochondrial compartments.

### Relevance to pathophysiology of metabolic and mitochondrial disease

Despite its sensitivity to high-sugar diet, *tko*^*25t*^ cannot be considered an exact model for diabetes in humans, since serum sugar levels are lower in the mutant than in wild-type flies (Fig 2E). The expression of several of the insulin-like peptides is slightly increased at the RNA level (Panel G in S4 Fig), but their exact functions in metabolic or growth regulation remain unclear, and mRNA levels may not reflect those of the peptides themselves [59]. Moreover, pAkt, considered a primary mediator of insulin signaling [20, 60], although down-regulated in *tko*^*25t*^ compared with wild-type (Panel F in S4 Fig), was still modestly increased in response to high-sugar diet (Panel F in S4 Fig).

Changes in insulin-signaling may contribute to lowered serum sugar (Fig 2E), enhanced expression of excretory sugar transporters (Fig 2A and 2C), decreased muscle biosynthesis (Sheets A and C in S4 Table) and increased triglycerides (Fig 3G), but these may be considered as adaptive responses to mitochondrial dysfunction rather than a pathological signature and, at least to some degree, are seen also in *tko*^*25t*^ larvae grown on zero-sugar medium.

Because it is also manifest on zero-sugar medium, the elevated level of triglycerides seen in *tko*^*25t*^ is not simply a classic ‘sugar-into-fat’ response to sugar overload. More likely, fat deposition represents an alternate system for storing energy needed to fuel metamorphosis, partially replacing the accumulation of muscle. Larval body wall muscle is largely turned over during metamorphosis [61], but *tko*^*25t*^ larvae exhibit down-regulation of mRNAs coding for structural proteins of muscle (Sheet A in S4 Table). The response of wild-type larvae to high-sugar diet involves up-regulation of muscle and cuticular components, as well as glycolysis and the cytosolic translation system (Sheet A in S4 Table). Unlike yeast or cancer cells, where high-glucose media promote more rapid growth [62], wild-type *Drosophila* larvae do not grow more rapidly on a high-sugar diet, possibly even slightly slower (Fig 1 and Panel A in S1 Fig). Increased biosynthesis of cuticle and muscle might be a signature of an avoidance response to a high-sugar environment. As already noted, sugar overload should be toxic to the epidermis and to the gut, due to protein glycation and/or NADPH depletion resulting from glucose oxidation. Increased production of cuticle proteins may protect the epidermis, whilst the accumulation of body wall muscle will facilitate the movement of the larva to a less stressful environment. The metabolic consequences of mitochondrial dysfunction may limit this response in *tko*^*25t*^ larvae. Muscle is also one of the most energy-consuming tissues, and developmental reprogramming that limits muscle biosynthesis, storing biomass for later catabolism during metamorphosis instead as fat, may be considered an energy-sparing response to ATP depletion that is signaled via AMPK or its downstream targets.

The metabolic crisis experienced by *tko*^*25t*^ larvae in high sugar diet may have a more general relevance in mitochondrial disease. Other data supports the idea that ketogenic or low carbohydrate diet is beneficial for mitochondrial disease patients or animal models thereof [63, 64]. The combination of ATP deletion and glycotoxic stress is likely to occur in many contexts relevant to human pathology. Although glucose homeostasis operates differently in mammals, some secretory tissues are naturally susceptible to glucose overload, notably the gut, and perhaps most importantly, the islet cells of the pancreas, which must respond to physiological fluctuations in glucose. *Drosophila* Gld does not have a strict orthologue in mammals: however, it is member of a multigene family of choline-glucose dehydrogenases that ultimately consume NADPH, whose many mammalian members remain functionally unidentified.

Our findings implicate pyruvate overload, ATP depletion and disturbed NADP/NADPH homeostasis as key elements of the metabolic crisis resulting from OXPHOS deficiency, especially in the presence of high dietary sugar, with deranged protein homeostasis in the secretory pathway as an unexpected outcome. As well as suggesting possible new drug targets in mitochondrial disease, they may also be relevant to cancer. To facilitate rapid growth, many tumors switch to aerobic glycolysis for ATP production (the Warburg effect), at the same time frequently down-regulating OXPHOS, commonly by accumulating mtDNA mutations. However, if what is true of *Drosophila* larvae is also true of such tumors, high glucose levels should provoke a metabolic crisis that curtails growth or may even lead to apoptosis, if NADPH/NADP+ homeostasis is pushed beyond a certain limit. Since many of the commonly used drugs in cancer chemotherapy are metabolized via the NADPH-consuming cytochrome P450 system, NADPH depletion should be considered a possible mechanism by which they are really provoking tumor destruction. In this case, tumors that become chemo-resistant via activation of multi-drug resistance pathway might still be vulnerable to glucose overload.

In humans, pyruvate treatment has been suggested to be effective in some cases of mitochondrial disease [65], whereas other studies found no convincing benefits from treatments such as with dichloroacetate, designed to increase mitochondrial pyruvate utilization and thus decrease cytosolic pyruvate load (for review see [66]). In *tko*^*25t*^ larvae, pyruvate supplementation is clearly deleterious, but dichloroacetate treatment also provided no benefit and tended to exacerbate rather than relieve developmental delay on both high and zero-sugar diets (Panel J in S4 Fig). Thus, the issue is not pyruvate levels *per se*, but the inability of OXPHOS-defective mitochondria to metabolize pyruvate. Therapies targeted on pyruvate might therefore cause more harm than good, by increasing pyruvate overload in the mitochondria.

## Experimental Procedures

### *Drosophila* stocks and maintenance

*tko*^*25t*^ [3] in the Oregon R background was as described previously [6]. *w*^*1118*^, standard balancers, gut-specific Kyoto GAL4 line 113094, and VDRC RNAi lines targeted on *Men* and *Gld* (#104016 and #108361, respectively) were obtained from stock centers. Except for use in specific experiments, flies were maintained at room temperature in plugged plastic vials containing standard high-sugar medium (see SI for full details), supplemented with 0.5% propionic acid (Sigma-Aldrich) and 0.1% (w/v) methyl 4-hydroxybenzoate (Nipagin, Sigma-Aldrich), then transferred to vials containing appropriate media for mating and larval culture in a 12 h light-dark cycle at 25°C, except where stated. To avoid selection of suppressors, *tko*^*25t*^ flies were maintained using FM7 balancer and homo/hemizygotes generated as needed for experiments. In general, to avoid any confounding effects from the balancer, wild-type flies in the same background were used as controls in parallel in all experiments. UAS-*tko*^*8*^ flies, described previously [22] were maintained in the *tko*^*25t*^ background, and their phenotype rechecked prior to experiments.

### Culture media

Fly food was created according to different recipes as detailed in SI. Most experiments used standard high-sugar medium [67], denoted HS, and ‘zero-sugar’ medium, denoted ZS, containing only agar, dried yeast extract, soya and maize flours, but no added sugars. Isocaloric media with lowered sugar content but other components in varying proportions were as indicated in S1 File. Various supplements, as indicated in figures and legends, were generally added from stock solutions after medium was cooled to 50°C, including sodium pyruvate or lactate (25 mg/ml), ornithine (5 or 20 μg/ml) and cycloheximide (up to 250 μg/ml). For tunicamycin (12 μM), low-melting point agarose was used instead of agar, wth cooling to 37°C before addition of drug.

### Developmental time and behavioral assays

Crosses were conducted in a minimum of 3, usually 4 or 5 replicates, and mean developmental time to eclosion (at 25°C, except where indicated), as well as bang-sensitivity were measured as described previously [15]. Standard deviations were calculated based on the distributions of mean eclosion day from replicate vials (except where indicated) or, for bang-sensitivity, from the distribution of recovery times from mechanical shock of all individual flies of a given sex and genotype tested (in batches of 30–50 flies). Larval feeding behaviour was assayed by dye ingestion. Briefly, individual larvae grown on HS or ZS medium were placed on petri dishes of the same medium containing 0.16% erioglaucine for 20 min, washed with PBS, dried, and then homogenized in 100 μl PBS. Homogenates were centrifuged at 12,000 g_max_ and dye uptake was measured spectrophotometrically (absorbance at 630 nm) using the supernatant fractions.

### Metabolite analysis

Steady-state levels of ATP, lactate and pyruvate were measured by enzyme-linked luminometry (ATP) or fluorometry in extracts from batches of 20 larvae or adult flies, using commercially available kits according to manufacturer’s protocols (Molecular Probes, Life Technologies for ATP, Abcam for pyruvate and lactate). Hemolymph extracts from batches of 30–50 larvae were treated overnight with trehalase (Sigma-Aldrich), following which total serum sugars were assayed using glucose (HK) reagent (Thermo Scientific). Triglyceride levels were measured essentially according to Tennesen et al. [68], using triglyceride reagent (Thermo Scientific), with subtraction of the background signal due to free glycerol. For global metabolite analysis, batches of 15 larvae were snap-frozen in liquid nitrogen, deproteinized by chloroform extraction, centrifugation and micro-filtration, then analyzed separately for anions and cations by CE-TOFMS in the presence of suitable standards. For full details see S1 File.

### Enzymatic analyses

Malic enzyme, glucose-6-phosphate dehydrogenase, 6-phosphogluconate dehydrogenase and isocitrate dehydrogenase were assayed essentially as described previously [69, 70, 71]. Isocitrate dehydrogenase and glutamate dehydrogenase activities were also analyzed separately using a commercially available IDH activity kit (Abcam), in order to evaluate the contribution of NAD^+^/NADH- and NADP^+^/NADPH-dependent isoforms to the total enzyme activity. See S1 File for full details.

### RNA analysis

For QRTPCR, RNA extraction from *Drosophila* adults and larvae, cDNA synthesis, PCR and data analysis were performed as described previously [67], using primer sets shown in S6 Table. For RNA-sequencing, RNA was extracted from flash-frozen batches of 30 larvae using miRNA Easy Mini Kit (Qiagen) and manufacturer’s instructions. Three biological replicate samples were produced by pooling 4 independent preparations to produce each replicate. RNA sequencing was performed on Hiseq 2500 sequencers (Illumina) using paired-end library and 100 bp read-legnth and otherwise standard protocols. Expression analysis was performed using Chipster. Sequencing reads were mapped to the *Drosophila* reference genome (BDGP release 5.72) using TopHat version 2.0.9, and differential expression analysis was performed using CuffDiff. The splicing pattern of Xbp1 was analysed by RT-PCR, PstI digestion and agarose gel electrophoresis [72].

### Protein analysis

Total protein was extracted from batches of 20 frozen larvae in phosphate buffer containing 150 mM NaCl, 1 mM EDTA, 2 M urea, 1.3% (w/v) SDS, 10 mg/ml each Complete protease inhibitor mix (Roche) and PhosSTOP phosphatase inhibitor mix (Roche), diluted into 4 x Laemmli loading buffer and (see S1 File for details) and electrophorsesed on Criterion TGX AnyKD precast SDS-PAGE gels (Bio-Rad). Protein was transferred to 0.45 μm Hybond ECL nitrocellulose membrane (Amersham, GE Heathcare Life Sciences). Blots were processed and visualized by standard methods (see S1 File for full details). Primary antibodies, used at 1:1,000 dilution, were against: Akt #4691 (Cell Signaling), phospho-Akt #4054 (Cell Signaling), AMPK #80039 (Abcam), phospho-AMPK #4188 (Cell Signaling), S6K #64804 (Abcam), phospho-S6K #9029 (Cell Signaling) and alpha-tubulin #52866 (Abcam, 1:10,000) with appropriate HRP-conjugated secondary antibodies (Vector Laboratories, 1:10,000): Horse Anti-Mouse IgG #PI-2000 or Goat Anti-Rabbit IgG #PI-1000. For further details see S1 File.

## Supporting Information

S1 Fig Supplementary data on modulation of *tko*^*25t*^ phenotype by diet. Time to eclosion of *tko*^*25t*^ and wild-type flies of sex as shown, grown on media of the indicated composition (see SI for details). In (A) asterisks denote significant differences from flies of the same genotype grown on 0% sucrose medium (Student’s *t* test, * showing *p* < 0.05, ** showing *p* < 0.01). For corresponding eclosion data of males see Fig 1A. In (B) and (E), horizontal lines indicate significant differences between flies of a given genotype, grown on different media (Student’s *t* test, * showing *p* < 0.05, ** showing *p* < 0.01). For corresponding eclosion data of males grown on high-sugar media, see Fig 4D. In (C) asterisks (**) denote significant difference from flies of the same genotype grown on all other media tested (Student’s *t* test, *p* < 0.01), which were not significantly different from each other. For corresponding eclosion data of males see Fig 1B. In (D) there were no significant differences from flies of the same genotype, grown on other media (Student’s *t* test, *p* > 0.05). In all experiments eclosion times for *tko*^*25t*^ flies were also significantly different from those of wild-type flies grown on the same medium (Student’s *t* test, *p* < 0.01). For corresponding eclosion data of males see Figs 1 and 4D.
(PDF)

S2 Fig Supplementary data on the ‘anti-sugar’ response of *tko*^*25t*^ flies. Expression levels of various genes, based on QRTPCR, in adult females of the indicated genotypes, grown on high-sugar medium. (A) Malpighian tubule-specific sugar transporters, (B) gut-specific α-glucosidases. All signals normalized to the levels in wild-type females. Horizontal bars denote values significantly different between genotypes (Student’s *t* test, * indicating *p* < 0.05, ** indicating *p* < 0.01).
(PDF)

S3 Fig Supplementary data on metabolite levels in *tko*^*25t*^ and wild-type flies. Relative levels of different metabolites in adult females or L3 larvae (as shown) of the indicated genotypes and growth conditions, based on (A) findings from enzyme-liked assays, (B) fluorescence spectrometry or (C, D) mass spectrometry. Absolute values are shown for (C) amino acids. Values in (A, B) are normalized to those for wild-type larvae grown on ZS medium, enabling them to be plotted alongside for comparison. A similar plot for those amino acids exhibiting substantial changes (here boxed in red) is shown in Fig 3D. Values in (D) for polyamines are normalized to the level of putrescine in wild-type larvae grown on ZS medium, enabling them to be plotted alongside for comparison. Absolute values from mass spectrometry are given in S1 Table. Horizontal bars denote significantly different data classes (Student’s *t* test, *p* < 0.05), except in (C), where significant differences in amino acid levels between wild-type and *tko*^*25t*^ are shown in Fig 3D, and presented in full in S7 Table.
(PDF)

S4 Fig Supplementary indicative data on dietary modulation of *tko*^*25t*^ phenotype. (A) Time to eclosion of female flies of the indicated genotypes and dietary conditions, on medium supplemented with pyruvate (pyr) or lactate (lact). In the presence of either supplement there were no significant difference in eclosion timing between *tko*^*25t*^ flies grown on high-sugar versus zero-sugar medium (Student’s *t* test, *p* > 0.05). See also Fig 4A. (B) Summary diagram of the major NADPH-producing enzymes. (C) Activities of the major NADPH-producing enzymes in extracts from *Drosophila* L3 larvae of the indicated genotypes and dietary conditions. (D, E) Time to eclosion of female flies of the indicated genotypes and dietary conditions, on medium supplemented (or not) with ornithine (orn), at the concentrations shown. * denotes value significantly different than for flies of the corresponding genotype and dietary condition, with ornithine *versus* without the supplement (Student’s *t* test, *p* < 0.05). (F) Western blots of extracts from L3 larvae of the indicated genotypes and dietary conditions, probed for AMPK, pAMPK (phosphorylated at Thr-172), Akt, pAkt (phosphorylated at Ser-505) or S6K, plus the α-tubulin loading control (αTub). See also Fig 4F. (G) QRTPCR of mRNAs for four of the *Drosophila* insulin-like peptide (dILP) genes, in larvae of the indicated genotype and dietary condition. Despite the trend, differences between genotypes were not significant for the dILP genes considered individually (Student’s *t* test, *p* > 0.05). (H, I) Analysis of Xbp1 splicing by RTPCR. (H) Agarose gel showing the product fragments diagnostic for the spliced (216S) and unspliced (239U) forms of Xbp1 mRNA (fragment sizes in bp). (I) Analysis by QRTPCR, in larvae of the indicated genotype and dietary condition, revealing only modest differences (all values normalized to those for wild-type larvae grown on high-sugar medium). (J) Time to eclosion of female flies of the indicated genotypes and dietary conditions, on medium supplemented with 12.5 mg/ml dichloroacetate (DCA). Males showed the same trends.
(PDF)

S5 Fig Supplementary data on effect of cycloheximide and tunicamycin on developmental timing of *tko*^*25t*^ and wild-type flies. (A, B) Repeats of experiment shown in Fig 5A, but using various ranges of cycloheximide concentrations. (A) Means ± SD of times to eclosion of flies of the sex and genotypes indicated, on media containing increasing amounts of cycloheximide. Based on pairwise *t* tests, and considering all the flies of a given sex and genotype cultured at a specific drug concentration as a single population, mean eclosion times were significantly different (*p* < 0.01) at different cycloheximide concentrations for *tko*^*25t*^ males or females at all doses tested, compared with flies grown on medium without drug, but the values for the different doses of drug tested were not different from each other. For control flies, values at all concentrations were significantly different from those without drug and from each other, except for 25 *versus* 75 μg/ml. (B) Means ± SD of times to eclosion of flies of the sex and genotypes indicated, on high-sugar medium, with or without cycloheximide (50 μg/ml). Based on pairwise *t* tests, and considering all the flies of a given sex and genotype cultured at a specific drug concentration as a single population, eclosion timed for flies cultured without drug were significantly different from those cultured with drug in each case (*p* < 0.01). (C) Pooled eclosion data from four independent experiments conducted with different concentration ranges of cycloheximide. Male developmental delay showed consistent decrease with increasing cycloheximide concentration. Females showed the same trend (Fig 5B). (D) Bang-sensitivity (recovery times) of *tko*^*25t*^ flies of the sexes indicated, grown on high-sugar medium with or without cycloheximide (150 μg/ml). Wild-type flies were not bang-sensitive. (E) Means ± SD of times to eclosion of flies of the sex and genotypes indicated, on high-sugar medium, with or without tunicamycin (12 μM). Asterisks denote significant differences between flies of a given sex and genotype cultured with or without drug (*p* < 0.01). A repeat experiment gave the same result.
(PDF)

S6 Fig Verification that Kyoto GAL4 driver line 113094 directs expression specifically in the gut. Micrographs of dissected L3 larvae expressing GFP driven by Kyoto GAL4 line 113094 (‘gut-GAL4)’, full genotypes as indicated, left-hand panels in visible light, right-hand panels showing green fluorescence. (A) nuclear-localized Stinger-GFP, (B) membrane-localized mCD8-GFP, (C) portion of top image from (B) at higher magnification, to show more detail of structures. As arrowed, GFP is expressed in the salivary glands (sg), gastric caecae (gc), foregut and mid-gut (mg), most strongly in its distal portion, but not in the imaginal discs (id), brain (b), hind-gut (hg), Malpighian tubule (mt), fat body (fb), proventriculus (p), or carcass (c). [Faint signal in carcass is background auto-fluorescence].
(PDF)

S1 File Supplemental information. Supplemental Materials and Methods, Supplemental References, legends to Supplemental Figures.
(PDF)

S1 Table Metabolome analysis; Sheet A: Results of Anion-Nucleotide; Sheet B: Results of Cation; Sheet C: Graphs of data from Sheet A; Sheet D: Graphs of data from Sheet B. (XLS)

S2 Table Output of RNAseq analysis: four pairwise comparisons at larval L3 stage, rank-ordered with no filtering. (XLS)

S3 Table Sheet A: Transcripts most reponsive to genotype (proportionate change in expression in c *tko*^*25t*^ ompared with wild-type)–Protein coding; Sheet B: Transcripts most reponsive to genotype (proportionate change in expression in *tko*^*25t*^ compared with wild-type)–Non-coding RNAs; Sheet C: Transcripts most reponsive to diet (proportionate change in expression in HS compared with ZS)–Protein coding; Sheet D: Transcripts most reponsive to diet (proportionate change in expression in HS compared with ZS)—Non-coding RNAs. (XLS)

S4 Table Sheet A: Genes regulated by genotype, absolute changes–Protein-coding genes; Sheet B: Genes regulated by genotype, absolute changes–Non-coding RNAs; Sheet C: Genes regulated by diet, absolute changes–Protein-coding genes; Sheet D: Genes regulated by diet, absolute changes–Non-coding RNAs. (XLS)

S5 Table Gene ontology analysis of transcriptomic data. (DOC)

S6 Table Oligonuceotide primers used for QRTPCR. (XLS)

S7 Table Statistical analysis of metabolome data. (XLS)
