# Correction: Mitochondrial Dysfunction Plus High-Sugar Diet Provokes a Metabolic Crisis That Inhibits Growth

**DOI:** 10.1371/journal.pone.0151421

**Published:** 2016-03-08

**Authors:** Esko Kemppainen, Jack George, Görkem Garipler, Tea Tuomela, Essi Kiviranta, Tomoyoshi Soga, Cory D. Dunn, Howard T. Jacobs

In the Funding section, the grant number from the funder EMBO (Installation Grant to CDD) is listed incorrectly. The correct grant number is: 2138.
