# Supplementary figures and images for: Mitochondrial Dysfunction Plus High-Sugar Diet Provokes a Metabolic Crisis That Inhibits Growth
Source: PLoS One. 2016 Jan 26;11(1):e0145836. doi: 10.1371/journal.pone.0145836 (PMC4728084; doi:10.1371/journal.pone.0145836)

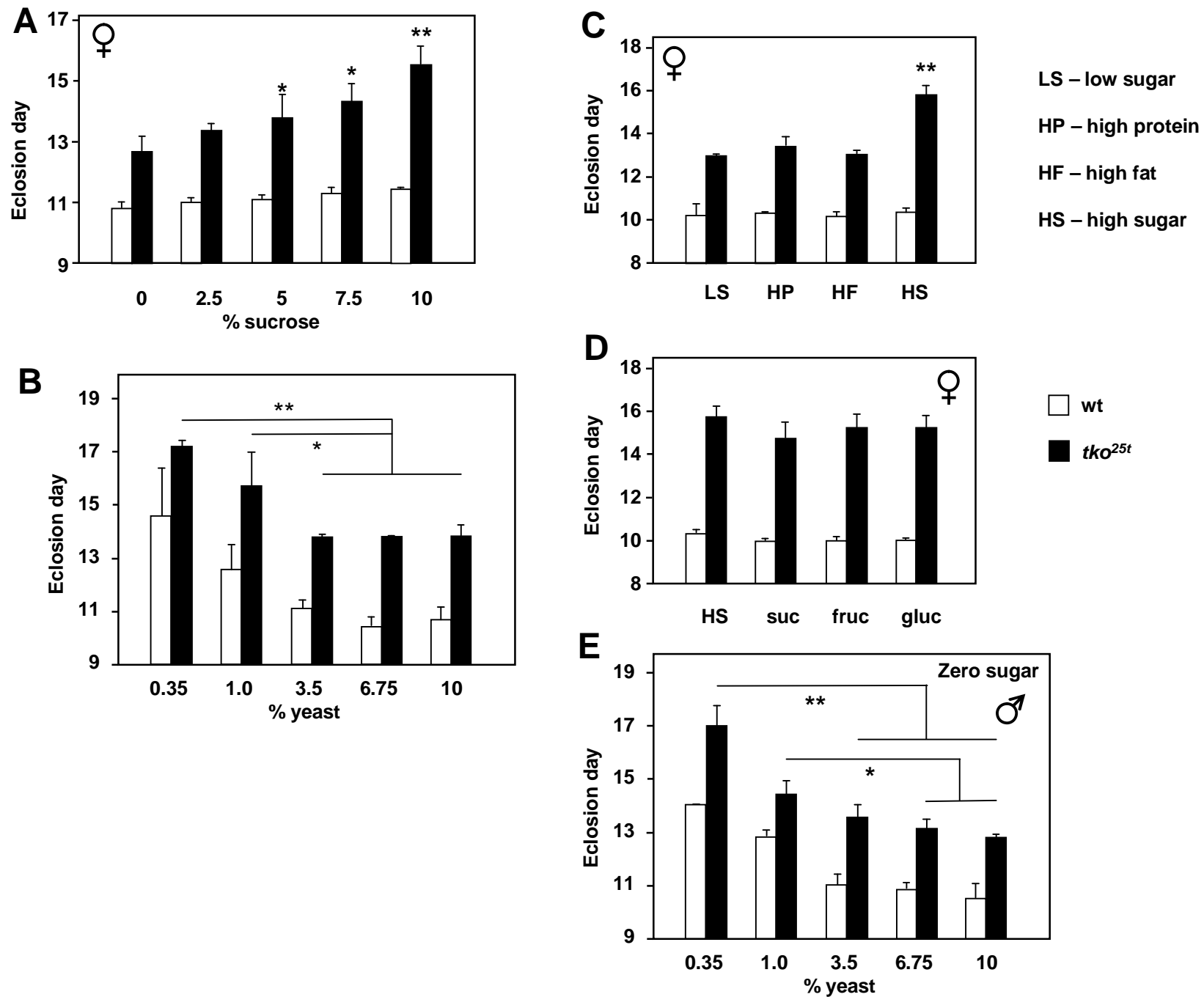

Figure S1, Kemppainen et al

Supplement: S1 Fig — Time to eclosion of tko25t and wild-type flies of sex as shown, grown on media of the indicated composition (see SI for details). In (A) asterisks denote significant differences from flies of the same genotype grown on 0% sucrose medium (Student’s t test, * showing p < 0.05, ** showing p < 0.01). For corresponding eclosion data of males see Fig 1A. In (B) and (E), horizontal lines indicate significant differences between flies of a given genotype, grown on different media (Student’s t test, * showing p < 0.05, ** showing p < 0.01). For corresponding eclosion data of males grown on high-sugar media, see Fig 4D. In (C) asterisks (**) denote significant difference from flies of the same genotype grown on all other media tested (Student’s t test, p < 0.01), which were not significantly different from each other. For corresponding eclosion data of males see Fig 1B. In (D) there were no significant differences from flies of the same genotype, grown on other media (Student’s t test, p > 0.05). In all experiments eclosion times for tko25t flies were also significantly different from those of wild-type flies grown on the same medium (Student’s t test, p < 0.01). For corresponding eclosion data of males see Figs 1 and 4D. (PDF) [file pone.0145836.s001.pdf]

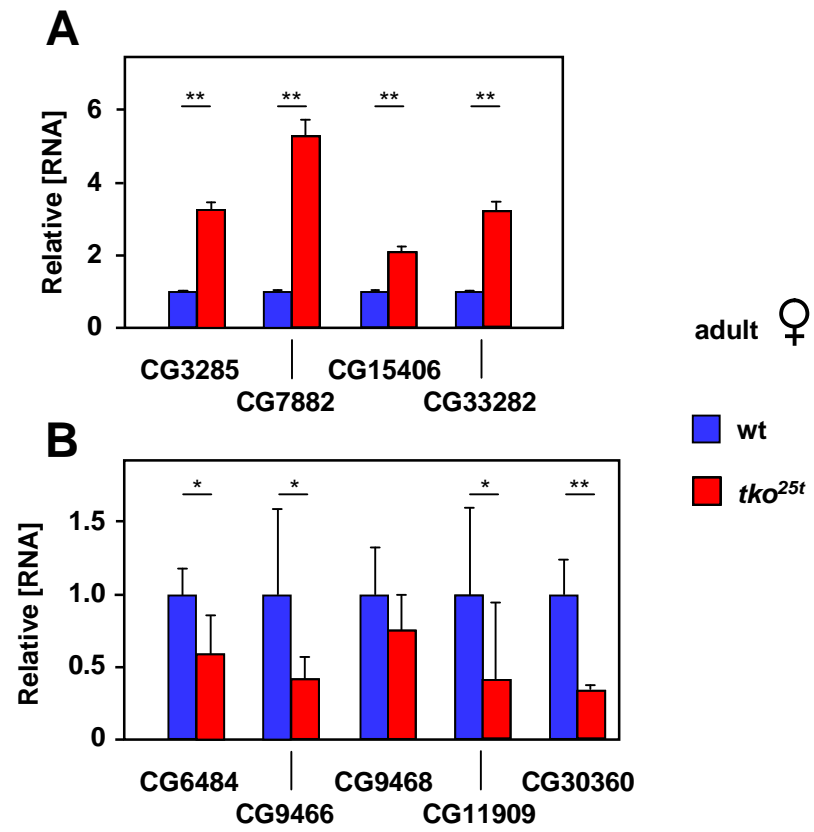

Figure S2, Kemppainen et al

Supplement: S2 Fig — Expression levels of various genes, based on QRTPCR, in adult females of the indicated genotypes, grown on high-sugar medium. (A) Malpighian tubule-specific sugar transporters, (B) gut-specific α-glucosidases. All signals normalized to the levels in wild-type females. Horizontal bars denote values significantly different between genotypes (Student’s t test, * indicating p < 0.05, ** indicating p < 0.01). (PDF) [file pone.0145836.s002.pdf]

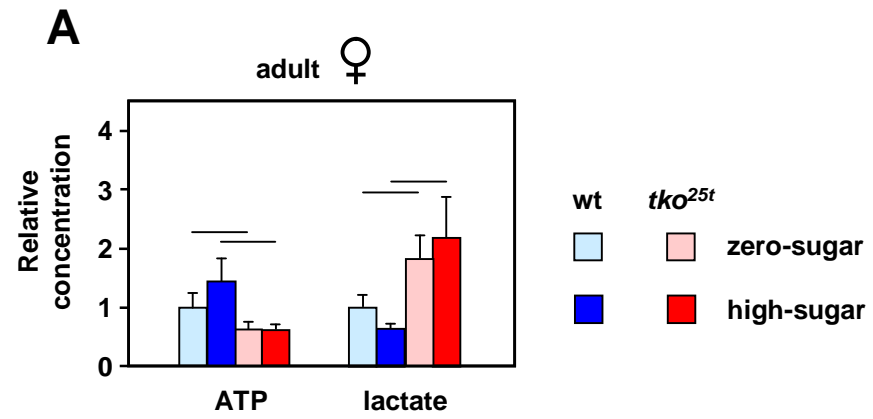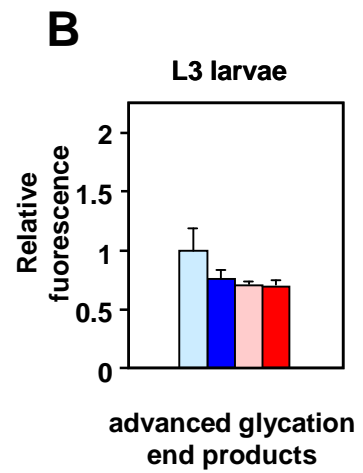

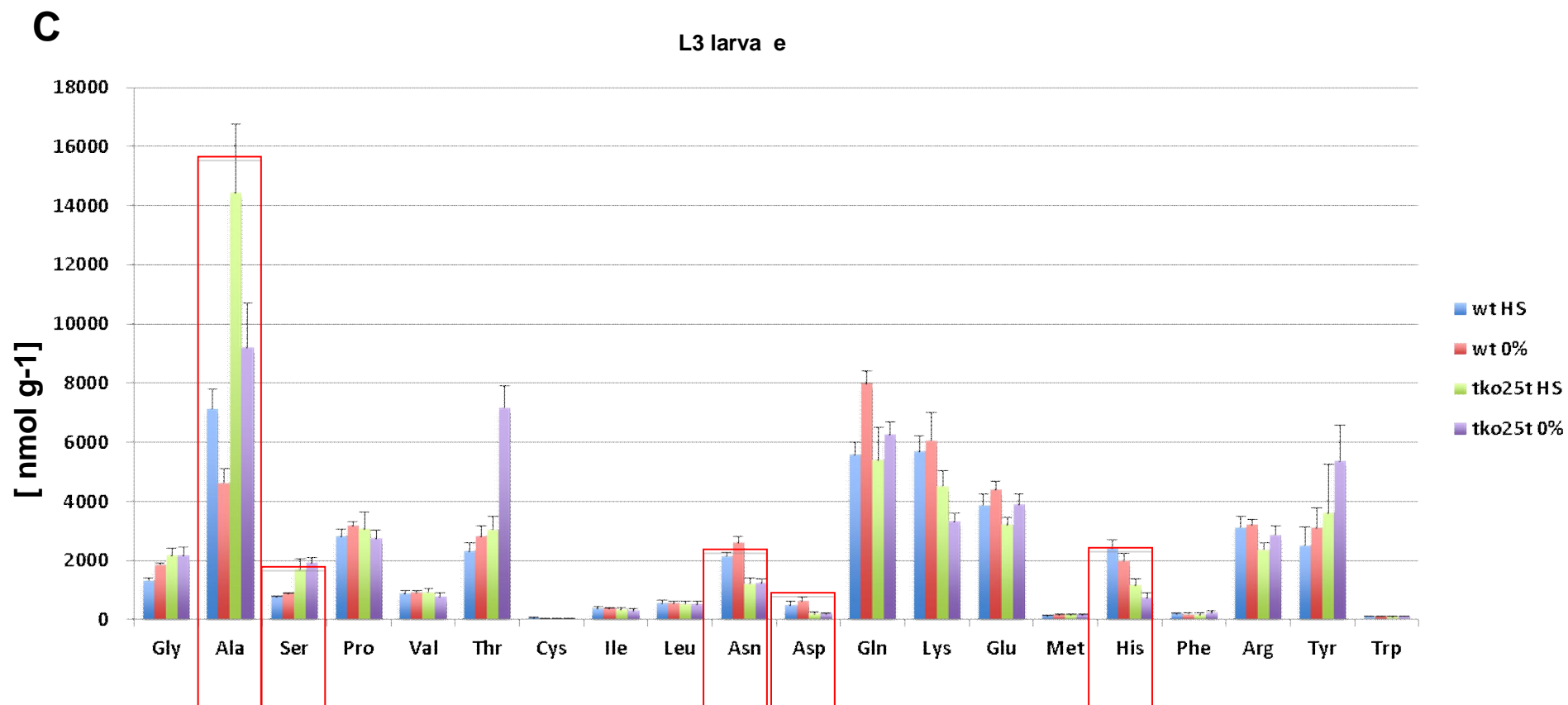

Figure S3, Kemppainen et al, page 2 of 3

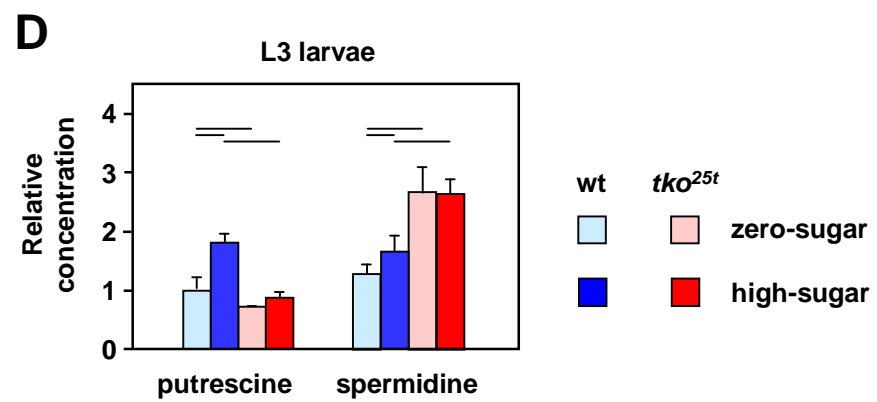

Supplement: S3 Fig — Relative levels of different metabolites in adult females or L3 larvae (as shown) of the indicated genotypes and growth conditions, based on (A) findings from enzyme-liked assays, (B) fluorescence spectrometry or (C, D) mass spectrometry. Absolute values are shown for (C) amino acids. Values in (A, B) are normalized to those for wild-type larvae grown on ZS medium, enabling them to be plotted alongside for comparison. A similar plot for those amino acids exhibiting substantial changes (here boxed in red) is shown in Fig 3D. Values in (D) for polyamines are normalized to the level of putrescine in wild-type larvae grown on ZS medium, enabling them to be plotted alongside for comparison. Absolute values from mass spectrometry are given in S1 Table. Horizontal bars denote significantly different data classes (Student’s t test, p < 0.05), except in (C), where significant differences in amino acid levels between wild-type and tko25t are shown in Fig 3D, and presented in full in S7 Table. (PDF) [file pone.0145836.s003.pdf]

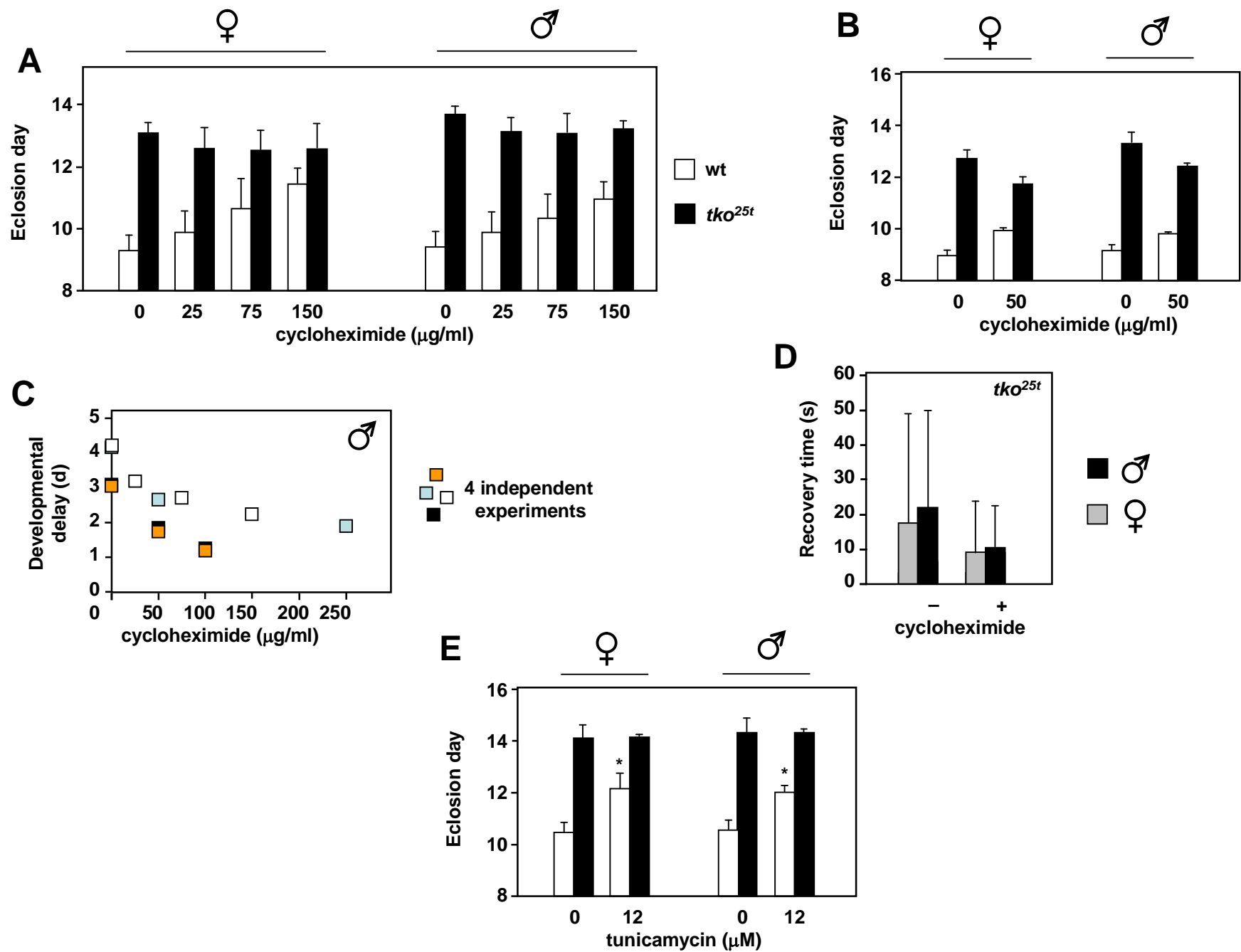

Figure S5, Kemppainen et al

Supplement: S5 Fig — (A, B) Repeats of experiment shown in Fig 5A, but using various ranges of cycloheximide concentrations. (A) Means ± SD of times to eclosion of flies of the sex and genotypes indicated, on media containing increasing amounts of cycloheximide. Based on pairwise t tests, and considering all the flies of a given sex and genotype cultured at a specific drug concentration as a single population, mean eclosion times were significantly different (p < 0.01) at different cycloheximide concentrations for tko25t males or females at all doses tested, compared with flies grown on medium without drug, but the values for the different doses of drug tested were not different from each other. For control flies, values at all concentrations were significantly different from those without drug and from each other, except for 25 versus 75 μg/ml. (B) Means ± SD of times to eclosion of flies of the sex and genotypes indicated, on high-sugar medium, with or without cycloheximide (50 μg/ml). Based on pairwise t tests, and considering all the flies of a given sex and genotype cultured at a specific drug concentration as a single population, eclosion timed for flies cultured without drug were significantly different from those cultured with drug in each case (p < 0.01). (C) Pooled eclosion data from four independent experiments conducted with different concentration ranges of cycloheximide. Male developmental delay showed consistent decrease with increasing cycloheximide concentration. Females showed the same trend (Fig 5B). (D) Bang-sensitivity (recovery times) of tko25t flies of the sexes indicated, grown on high-sugar medium with or without cycloheximide (150 μg/ml). Wild-type flies were not bang-sensitive. (E) Means ± SD of times to eclosion of flies of the sex and genotypes indicated, on high-sugar medium, with or without tunicamycin (12 μM). Asterisks denote significant differences between flies of a given sex and genotype cultured with or without drug (p < 0.01). A repeat experiment ga [file pone.0145836.s005.pdf]

**A**

**w<sup>-</sup> ; UAS-mCD8-GFP/*gut-GAL4* ; +**

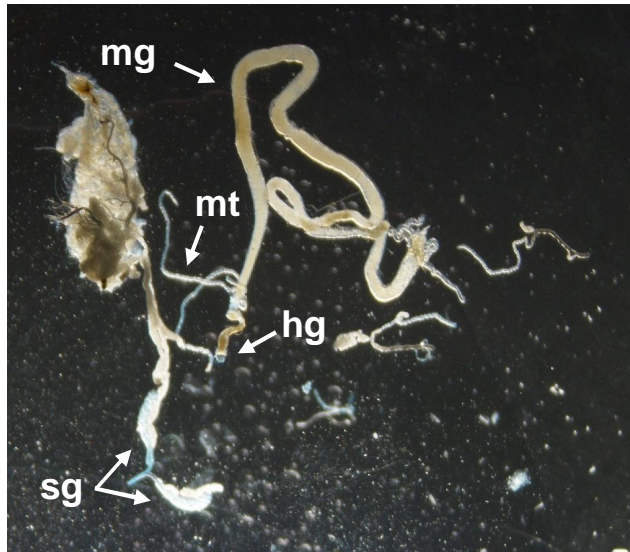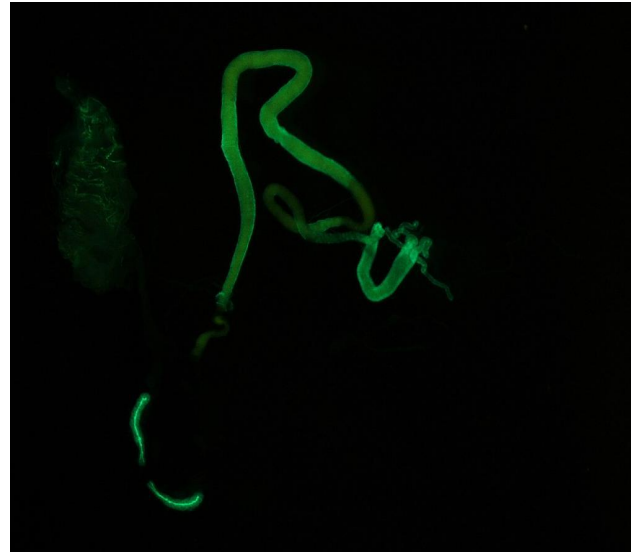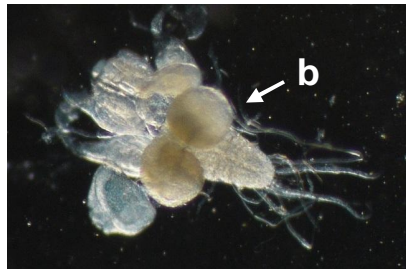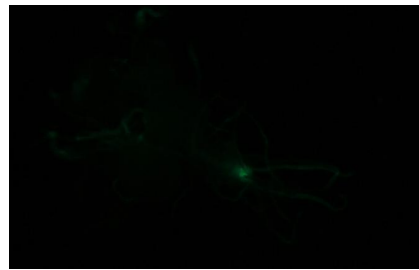

**B** *w<sup>-</sup> ; UAS-Stinger-GFP/*gut-GAL4* ; +*

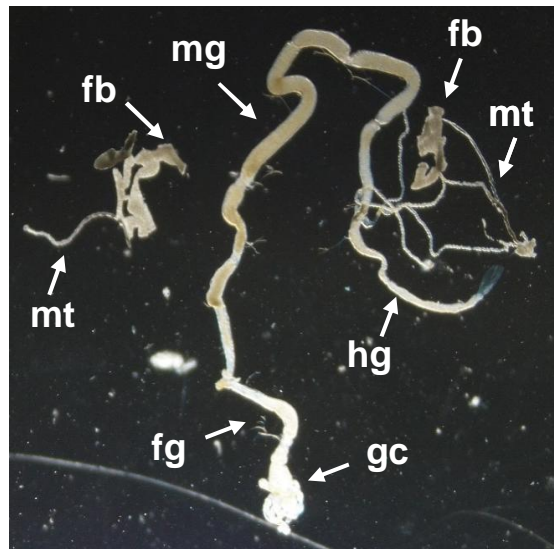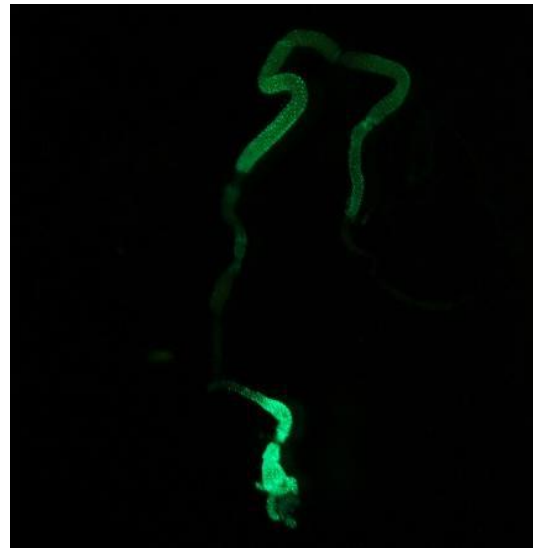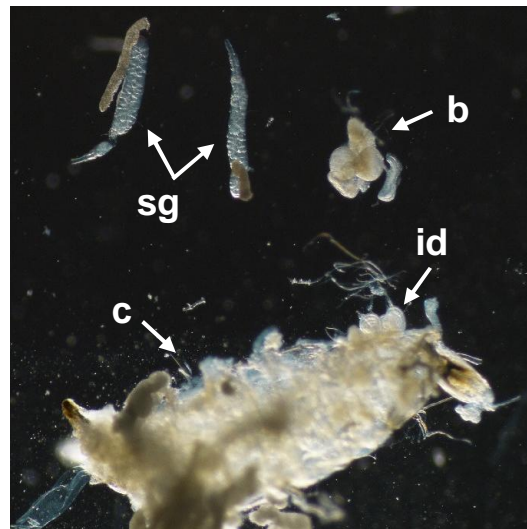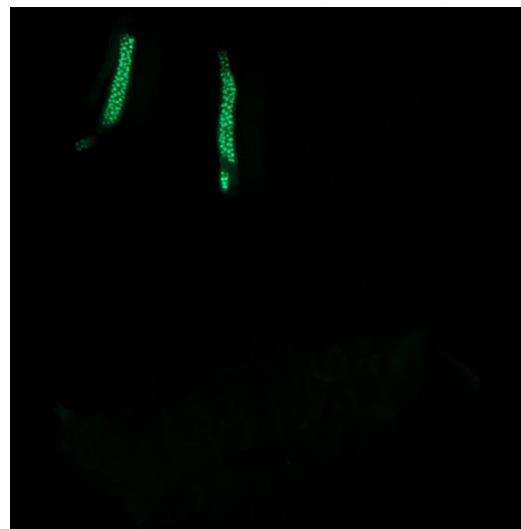

**C**

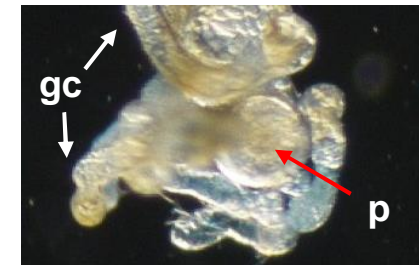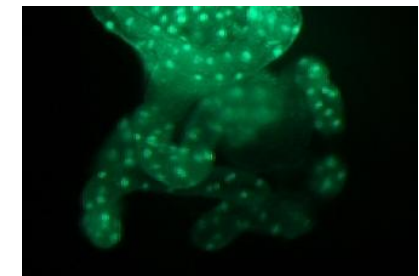

Supplement: S6 Fig — Micrographs of dissected L3 larvae expressing GFP driven by Kyoto GAL4 line 113094 (‘gut-GAL4)’, full genotypes as indicated, left-hand panels in visible light, right-hand panels showing green fluorescence. (A) nuclear-localized Stinger-GFP, (B) membrane-localized mCD8-GFP, (C) portion of top image from (B) at higher magnification, to show more detail of structures. As arrowed, GFP is expressed in the salivary glands (sg), gastric caecae (gc), foregut and mid-gut (mg), most strongly in its distal portion, but not in the imaginal discs (id), brain (b), hind-gut (hg), Malpighian tubule (mt), fat body (fb), proventriculus (p), or carcass (c). [Faint signal in carcass is background auto-fluorescence]. (PDF) [file pone.0145836.s006.pdf]
