# Supplementary material for: Mitochondrial Dysfunction Plus High-Sugar Diet Provokes a Metabolic Crisis That Inhibits Growth
Source: PLoS One. 2016 Jan 26;11(1):e0145836. doi: 10.1371/journal.pone.0145836 (PMC4728084; doi:10.1371/journal.pone.0145836)

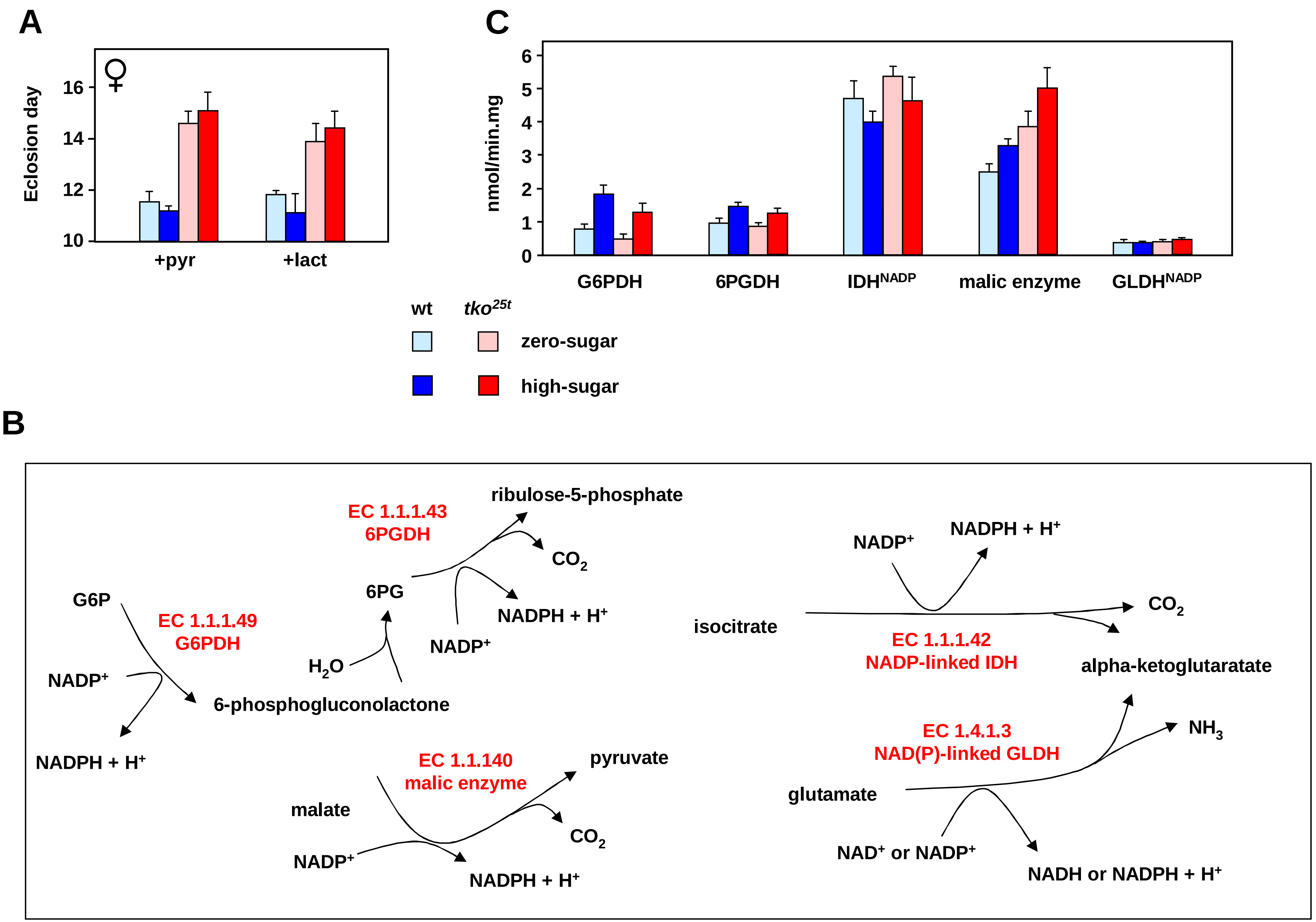

Figure S4, Kemppainen et al, page 1 of 3

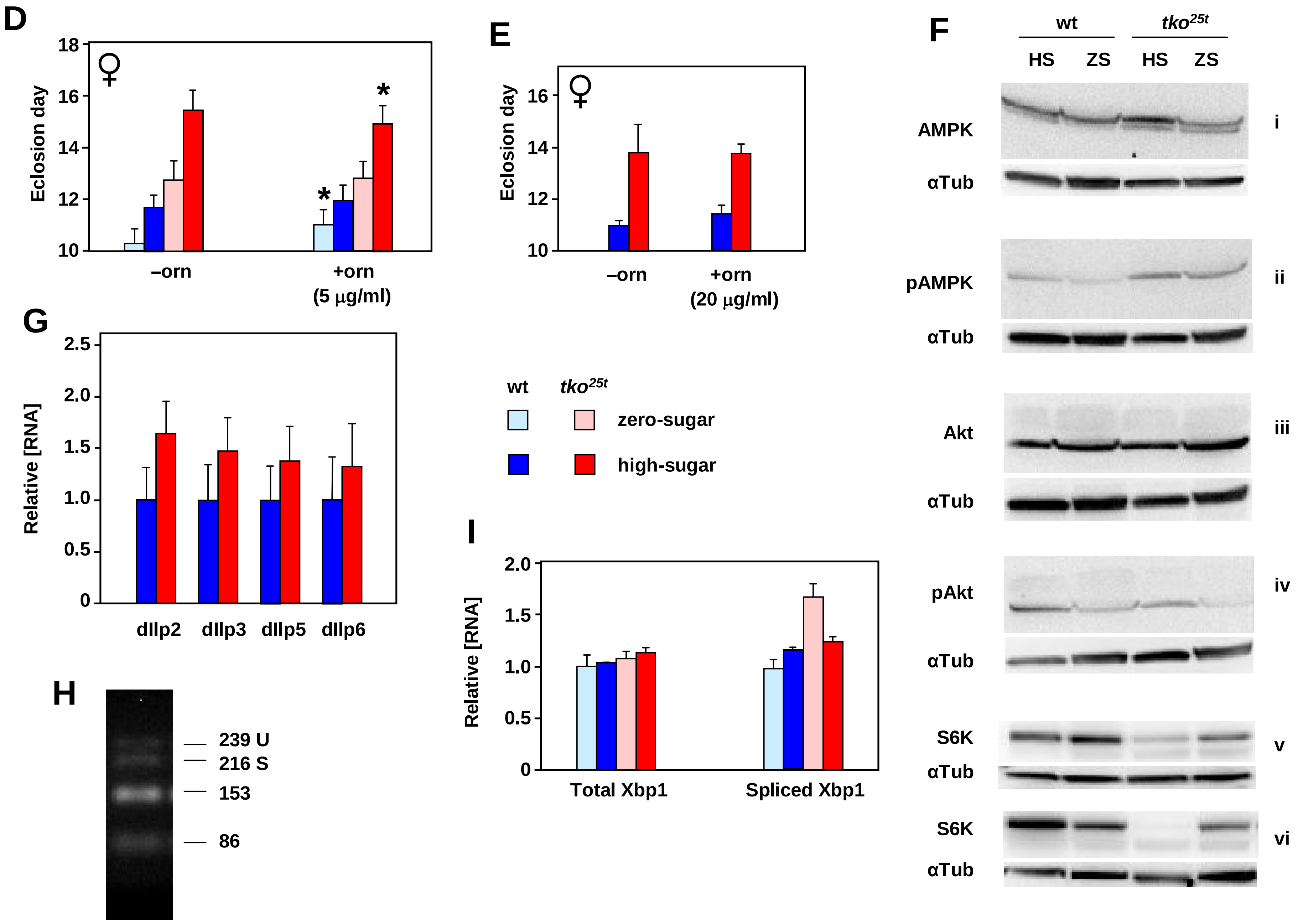

Figure S4, Kemppainen et al, page 2 of 3

J

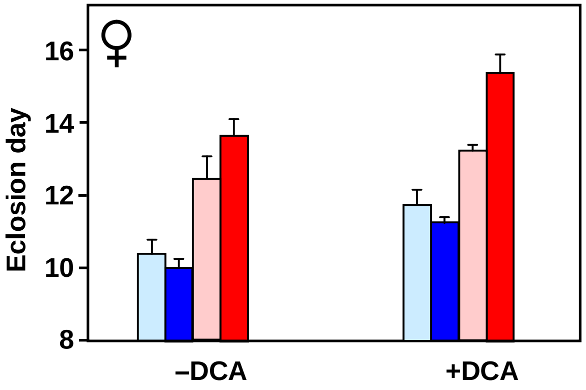

Supplement: S4 Fig — (A) Time to eclosion of female flies of the indicated genotypes and dietary conditions, on medium supplemented with pyruvate (pyr) or lactate (lact). In the presence of either supplement there were no significant difference in eclosion timing between tko25t flies grown on high-sugar versus zero-sugar medium (Student’s t test, p > 0.05). See also Fig 4A. (B) Summary diagram of the major NADPH-producing enzymes. (C) Activities of the major NADPH-producing enzymes in extracts from Drosophila L3 larvae of the indicated genotypes and dietary conditions. (D, E) Time to eclosion of female flies of the indicated genotypes and dietary conditions, on medium supplemented (or not) with ornithine (orn), at the concentrations shown. * denotes value significantly different than for flies of the corresponding genotype and dietary condition, with ornithine versus without the supplement (Student’s t test, p < 0.05). (F) Western blots of extracts from L3 larvae of the indicated genotypes and dietary conditions, probed for AMPK, pAMPK (phosphorylated at Thr-172), Akt, pAkt (phosphorylated at Ser-505) or S6K, plus the α-tubulin loading control (αTub). See also Fig 4F. (G) QRTPCR of mRNAs for four of the Drosophila insulin-like peptide (dILP) genes, in larvae of the indicated genotype and dietary condition. Despite the trend, differences between genotypes were not significant for the dILP genes considered individually (Student’s t test, p > 0.05). (H, I) Analysis of Xbp1 splicing by RTPCR. (H) Agarose gel showing the product fragments diagnostic for the spliced (216S) and unspliced (239U) forms of Xbp1 mRNA (fragment sizes in bp). (I) Analysis by QRTPCR, in larvae of the indicated genotype and dietary condition, revealing only modest differences (all values normalized to those for wild-type larvae grown on high-sugar medium). (J) Time to eclosion of female flies of the indicated genotypes and dietary conditions, on medium supplemented with 12.5 mg/ml dichloroacetate (DCA). Males showed t [file pone.0145836.s004.pdf]
