# Supplementary material for: Mitochondrial Dysfunction Plus High-Sugar Diet Provokes a Metabolic Crisis That Inhibits Growth
Source: PLoS One. 2016 Jan 26;11(1):e0145836. doi: 10.1371/journal.pone.0145836 (PMC4728084; doi:10.1371/journal.pone.0145836)
Supplement: S1 File — Supplemental Materials and Methods, Supplemental References, legends to Supplemental Figures. (PDF) [file pone.0145836.s007.pdf]

Mitochondrial dysfunction plus high-sugar diet provokes a metabolic crisis that inhibits growth

Esko Kemppainen, Jack George, Görkem Garipler, Tea Tuomela,

Essi Kiviranta, Tomoyoshi Soga, Cory D. Dunn & Howard T. Jacobs

SUPPLEMENTAL INFORMATION

SUPPLEMENTAL EXPERIMENTAL PROCEDURES

Culture media for Drosophila

Fly food recipes, using the same nomenclature as in the main text, were as follows, always in 1 % agar (Oriola), and with all ingredients shown as w/v:

HS (standard high-sugar medium) – 3% glucose, 1.5% sucrose, 3% treacle (Tate & Lyle, UK), 3.5% dried yeast, 1% soya flour, 1.5% maize flour

ZS (‘zero-sugar’ medium) – 3.5% dried yeast, 1% soya flour, 1.5% maize flour

Yeast series – HS or ZS medium, but with variable yeast content, from zero to 10% (see Fig. 1, S1)

Sugar series – ZS medium but with variable sucrose content, from zero to 10% (see Fig. 1, S1)

Isocaloric media with decreased sugar and increased protein and/or fat content: LS (low sugar) medium – 5.5% dried yeast, 2.5% maize flour, 2.5% soya flour, 3% treacle; HP (high protein) medium – 5.5% dried yeast, 1% maize flour, 1% soya flour, 3% soya protein, 3% treacle; HF (high fat) medium – 5.5% dried yeast, 1.5% maize flour, 1.5% soya flour, 0.75% olive oil, 3% treacle.

Sugar type series – HS medium but with all of the sugars as sucrose, fructose or glucose (denoted in Fig. S1 as suc, fruc and gluc, respectively)

Metabolite analysis by CE-TOFMS

Batches of 20 larvae or adult flies were homogenized in 100 µl of 6 M guanidine hydrochloride on ice. The homogenate was incubated at 95 °C for 5 min and centrifuged at 12,000 g_{max} for 5 min at 4 °C. The supernatants were transferred to fresh tubes and stored at -80 °C, then diluted 1:10 with PBS (pH 7.4) for analysis. Steady-state ATP levels were determined using an ATP determination kit (Molecular Probes, Life Technologies) according to manufacturer’s protocol, in which 90 µl of the ATP reaction mix was combined with 10 µl of the diluted sample to initiate the reaction and luminescence was measured immediately at

535 nm using a Hidex Chameleon plate reader. ATP standards were used to generate a standard curve and ATP concentrations were normalized to soluble protein in the samples, measured with the Bradford assay. Pyruvate and lactate were measured using fluorescence-based determination kits (Abcam) according to manufacturer's instructions. To 10 µl of sample was added 50 µl of either lactate or pyruvate reaction mix. Reactions were incubated at room temperature for 30 min after which fluorescence was measured (excitation at 535 nm, emission at 590 nm) using a plate reader. Lactate and pyruvate standards were used to generate standard curves and concentrations were normalized to soluble protein as above.

For determination of total serum sugars, batches of 30-50 freshly collected larvae were rinsed with PBS, dried and transferred into a 0.2 ml vial with small holes in the bottom. Larvae were then punctured several times with a 27 gauge needle and the small vial inserted inside of a larger vial. Larvae were then centrifuged at 1500 g_{max} for 5 min at 4 °C in order to drain hemolymph. Aliquots of 2 µl of hemolymph were transferred into fresh vials containing 8 µl of ice-cold TBS (pH 6.6), and incubated at 70 °C for 5 min, then centrifuged at 12,000 g_{max} for 1 min at RT. Supernatants were transferred into fresh vials and stored frozen at -20°C until analysis. Three µl of hemolymph extract were incubated with 0.5 µg of porcine kidney trehalase (Sigma-Aldrich) overnight at 37 °C, then mixed with 150 µl of glucose (HK) reagent (Thermo Scientific). Reactions were incubated at 37 °C for 1 h and absorbance at 530 nm was measured with a Hidex Chameleon plate reader. Glucose standards were used to determine the absolute glucose levels in the samples. Glucose levels were normalized to the amount of soluble protein in the samples, as determined using the Bradford assay. A minimum of eight replicate samples per genotype and dietary condition were used.

Triglyceride levels were measured essentially according to Tennessen et al (2014). Batches of 20 larvae were collected, rinsed several times with PBS in a 1.5 ml microcentrifuge tube and snap-frozen in liquid nitrogen. Frozen larvae were homogenized in 100 µl of ice-cold PBS containing 0.05% Tween 20. A 10 µl aliquot of the homogenate was diluted 1:10 with PBS and set aside to measure protein content with the Bradford assay. The larval lysate was incubated at 70 °C in order to inactivate endogenous lipases. One 20 µl aliquot of the heat-

treated lysate was mixed with 20 µl of triglyceride reagent (Thermo Scientific) and another with 20 µl of PBS. Both reactions were then incubated at 37 °C for 1 h. After this, 100 µl of free glycerol reagent (Sigma) was added to each sample. Samples were mixed well, incubated at 37 °C for 5 min and finally centrifuged for 3 min at full speed. 100 µl of the supernatant was used to measure absorbance at 540 nm. Glycerol solution (0.26 mg/ml in PBS, equaling 2.5 mg/ml of triolein) was used to generate a standard curve. Triglyceride content of each sample was determined by subtracting the glycerol content of the untreated sample from the triglyceride reagent-treated aliquot. For global metabolite analysis, batches of 15 mid-third instar larvae were harvested, washed with PBS and snap-frozen in liquid nitrogen. Frozen samples were completely homogenized by a cell disrupter (Shake Master NEO; Bio Medical Science, Tokyo, Japan) at 4°C, after adding 500 µl of methanol containing internal standards [20 µM each of methionine sulfone and 2-(N-morpholino)-ethanesulfonic acid (MES) and D-camphol-10-sulfonic acid (CSA)]. The homogenate was then mixed with 200 µl of Milli-Q water and 500 µl of chloroform and centrifuged at 9,100 g_{max} for 4 h at 4 °C, after which the aqueous solution was centrifugally filtered through a 5-kDa cut-off filter (Millipore) to remove proteins. The filtrate was centrifugally concentrated and dissolved in 50 µl of water containing reference compounds (200 µM each of 3-aminopyrrolidine and trimesate). Prior to CE-TOFMS analysis, the sample solution for anion was diluted five times and that for cation was diluted 10 times with water.

In all CE-TOFMS experiments, an Agilent CE capillary electrophoresis system (Agilent Technologies, Waldbronn, Germany), an Agilent 6220 LC/TOFMS system (Agilent Technologies, Palo Alto, CA), an Agilent 1100 series binary HPLC pump, a G1603A Agilent CE-MS adapter and a G1607A Agilent CE-ESI-MS sprayer kit were used. Data were acquired with the G2201AA Agilent ChemStation software for CE and Agilent MassHunter software.

Cationic metabolites were separated in a fused silica capillary (50 μm i.d. × 100 cm) filled with 1 M formic acid as the electrolyte. A sample solution was injected at 50 mbar for 5 s (5 nl) and a voltage of 30 kV was applied. The capillary temperature and sample tray were set at 20 °C and below 5° C, respectively. Methanol/water (50% v/v) containing 0.1 μM hexakis(2,2-difluoroethoxy) phosphazene (Hexakis) was delivered as the sheath liquid at 10 μl/min. ESI-TOFMS was performed in the positive ion mode, and the capillary voltage was set at 4 kV. The flow rate of heated dry nitrogen gas (heater temperature, 300 °C) was maintained at 7 psig. At TOFMS, the fragmentor, skimmer and Oct RFV voltages were set at 75, 50 and 125 V, respectively. Automatic recalibration of each acquired spectrum was achieved using the masses of reference standards ([^{13}C isotopic ion of a protonated methanol dimer (2MeOH+H)]$^+$, *m/z* 66.0632) and ([hexakis +H]$^+$, *m/z* 622.0290). Exact mass data were acquired at a rate of 1.5 spectra/s over a 50–1,000 *m/z* range (Soga et al, 2006).

For anionic metabolite analysis, the original Agilent SST316Ti stainless steel ESI needle was replaced with an Agilent G7100-60041 platinum ESI needle (Soga et al. 2009). Commercially available COSMO(+), chemically coated with cationic polymer, capillary (50 μm i.d. x 105 cm) (Nacalai Tesque, Kyoto, Japan) were used as the separation capillary. A 50 mM ammonium acetate solution (pH 8.5) was the electrolyte for CE separation (Soga et al, 2002). A sample solution was injected at 50 mbar for 30 s (30 nl) and a voltage of -30 kV was applied. Methanol/5 mM ammonium acetate (50% v/v) containing 0.1 μM hexakis was delivered as the sheath liquid at 10 μl/min. ESI-TOFMS was performed in the negative ion mode, and the capillary voltage was set at 3.5 kV. At TOFMS, the fragmentor, skimmer and Oct RFV voltages were set at 100, 50 and 200 V, respectively. Automatic recalibration of each acquired spectrum was performed using reference masses of reference standards ([13C isotopic ion of deprotonated acetic acid dimer (2CH3COOH-H)]$^-$, m/z 120.03834), and ([Hexakis + deprotonated acetic acid (CH3COOH-H)]$^-$, m/z 680.03554). Other conditions are the same as in cationic metabolite analysis.

Ezymatic analyses

Enzymatic assays were adapted from (Hinman & Blass 1981), (Merritt et al. 2005) and (Merritt et al. 2009). 20 frozen larvae were homogenized on ice in 200 µl of cold 100 mM Tris-HCl (pH 7.4). The reaction conditions for each enzyme were as follows (all at pH 7.4): malic enzyme activity was assayed in the presence of 100 mM Tris-HCl, 34 mM NADP, 50 mM $MnCl_2$, 50 mM DL-malic acid; glucose-6-phosphate dehydrogenase was assayed in the presence of 100 mM Tris-HCl, 0.34 mM NADP, 3.5 mM D-glucose-6-phosphate; 6-phosphogluconate dehydrogenase was assayed in the presence of 100 mM Tris-HCl, 0.34 mM NADP, 3.5 mM 6-phosphogluconate; isocitrate dehydrogenase was assayed in the presence of 100 mM Tris-HCl, 0.34 mM NADP, 0.84 mM $MgCl_2$, 1.37 mM DL-isocitric acid. Enzyme inhibitors sodium gluconate, sodium pyruvate and sodium lactate were used at 100 mM final concentration, where indicated. Reactions were initiated by combining 10 µl of the larval homogenate with 90 µl of each reaction mixture. Increase in absorbance at 510 nM was detected in the presence of 0.6 mM iodonitrotetrazolium chloride and 6.5 µM phenazine methosulfate. Absorbance was measured at 1-2 min intervals for 15 min and NADPH standards were used to transform absorbance values into changes in NADPH concentration. Enzyme activity was normalized to total soluble protein, measured with the Bradford assay. Isocitrate dehydrogenase and glutamate dehydrogenase activities were analyzed separately using a commercially available IDH activity kit (Abcam), in order to evaluate the contribution of NAD^+/NADH and $NADP^+$/NADPH dependent isoforms to the total enzyme activity. For this, 20 frozen larvae were homogenized as before in 200 µl of cold manufacturer's IDH assay buffer. Enzyme activities of IDH and GDH were analyzed using manufacturer's IDH substrate and 100 mM L-glutamate as substrates, respectively, and either NAD^+ or $NADP^+$ as co-factors. Reactions were initiated by combining 50 µl of the reaction mix with 10 µl of sample. Absorbance measurement (at 450 nm), enzyme activity determination and normalization were carried out as before.

RNA analysis

For RNA-sequencing, RNA was extracted from flash-frozen batches of 30 larvae using miRNA Easy Mini Kit (Qiagen) and manufacturer's instructions. Three biological replicate samples were produced by pooling 4 independent preparations to produce each replicate. RNA sequencing was performed on Hiseq 2500 sequencers (Illumina) using paired-end library and 100 bp read-legnth and otherwise standard protocols. Expression analysis was performed using Chipster. Sequencing reads were mapped to the *Drosophila* reference genome (BDGP release 5.72) using TopHat version 2.0.9, and differential expression analysis was performed using CuffDiff. The splicing pattern of Xbp1 was analysed by RT-PCR, PstI digestion and agarose gel eletrophoresis, according to Park et al (2014b).

For QRTPCR, RNA extraction from *Drosophila* adults and larvae, cDNA synthesis, PCR and data analysis were performed essentially as described previously (Fernandez-Ayala et al, 2009), using primer sets shown in Table S4. Briefly, 100 mg of flies or larvae (approx. 30 females, 40 males or 25 larvae) were homogenized in 1 ml of TRIzol-reagent (Life Technologies). The homogenate was incubated at room temperature for 5 min after which 200 µl of chloroform was added. After incubation for a further 3 min at room temperature samples were centrifuged at 12,000 g_{max} for 10 min at 4 °C. After transfer of 600 µl of the upper (aqueous) phase to a fresh tube, RNA was precipitated for 10 min at room temperature by adding an equal volume of isopropanol. RNA was polluted by centrifugation at 12,000 g_{max} for 10 min at 4 °C, washed with 75% ethanol in DEPC/treated water and resuspended in 200 µl of DEPC-treated water. Prior to cDNA synthesis, RNA samples were treated with 0.05 U/ml DNase I (Thermo Scientific) in DNase I buffer (100 mM Tris-HCl, 25 mM $MgCl_2$, 1 mM $CaCl_2$) at 37 °C for 1 h. RNA was recovered by phenol:chloroform extraction, ethanol precipitation and resuspension in DEPC-treated water. One µg of RNA was used for cDNA synthesis using the High-Capacity cDNA synthesis kit (Life Technologies) and manufacturer's instructions. Two replicate reactions of each sample were pooled and diluted 1:20 with water, from which 2 µl was used for quantitative real-time PCR (QRTPCR) performed using Fast SYBR Green Master Mix (Life Technologies) and 0.5 mM final primer

concentrations (primers listed in Table S4). Triplicate QRTPCR reactions were performed and data was acquired using a StepOne Plus instrument and software (Life Technologies). Expression levels were normalized against that of the housekeeping gene *RpL32*.

Protein analysis

Total protein was extracted from batches of 20 frozen larvae. Three replicate samples per condition per experiment were pooled to produce a single individual sample for analysis. All analyses were repeated independently at least three times. Larvae were homogenized in 200 μl of lysis buffer containing 150 mM NaCl, 1 mM EDTA, 2 M urea, 1.3 % (w/v) SDS, 10 mg/ml each Complete protease inhibitor mix (Roche) and PhosSTOP phosphatase inhibitor mix (Roche). Homogenates were centrifuged for 1 min at 12,000 g_{max} at 4 °C, after which supernatants were mixed with one-third volume of 4 x Laemmli loading buffer (40% (w/v) glycerol, 8% (w/v) SDS, 250 mM Tris-HCl (pH 6.8), 0.015% (w/v) bromophenol blue and 200 mM DTT), heated for 7 min at 95 °C and cooled on ice. Approximately 30 µg of protein was loaded on Criterion TGX AnyKD precast SDS-PAGE gels (Bio-Rad) and run in ProSieve EX Running buffer (Lonza). Protein was transferred to 0.45 µm Hybond ECL nitrocellulose membrane (Amersham, GE Heathcare Life Sciences) in ProSieve EX Transfer Buffer (Lonza) using Criterion blotters (Bio-Rad). Membranes were blocked at room temperature for 1 h in either TBS-Tween with 2.5% (w/v) dried nonfat milk or with PBS-Tween with 2.5% (w/v) BSA. Primary antibody incubations were carried out overnight at 4 °C in PBS-Tween with 2.5% (w/v) BSA. Membranes were then washed 3 x 5 min in PBS-Tween and incubated with HRP-conjugated secondary antibody for 1 h at room temperature in PBS-Tween with 2.5% (w/v) dried nonfat milk. Unbound secondary antibody was removed by three 5 min washes with PBS-Tween and finally once in PBS. Protein was detected and visualized using SuperSignal West Femto Chemiluminescent Substrate (Pierce, Thermo Scientific) and ChemiDoc imager (Bio-Rad). Primary antibodies used were: Anti-Akt #4691 (Cell Signaling), Anti-phospho-Akt #4054 (Cell Signaling), anti-AMPK #80039 (Abcam), anti-phospho-AMPK #4188 (Cell Signaling), anti-S6K #64804 (Abcam) and anti-phspho-S6K

#9029 (Cell Signaling). The HRP-conjugated secondary antibodies were Peroxidase Horse Anti-Mouse IgG #PI-2000 (Vector Labs) and Peroxidase Goat Anti-Rabbit IgG #PI-1000 (Vector Labs). Primary and secondary antibodies were used at 1:1,000 and 1:10,000 dilutions, respectively. Alpha-Tubulin #52866 (Abcam) was used as loading control at 1:10,000 dilution. Equal loading was also confirmed post-analysis by Ponceau-S staining.

SUPPLEMNTAL REFERENCES

Soga, T., Baran, R., Suematsu, M., Ueno, Y., Ikeda, S., Sakurakawa, T., Kakazu, Y., Ishikawa, T., Robert, M., Nishioka, T. and Tomita, M. (2006) Differential metabolomics reveals ophthalmic acid as an oxidative stress biomarker indicating hepatic glutathione consumption. J. Biol. Chem. 281, 16768-167676.

Soga, T., Igarashi, K., Ito, C., Mizobuchi, K., Zimmermann, H.P. and Tomita, M. (2009) Metabolomic profiling of anionic metabolites by capillary electrophoresis mass spectrometry. Anal. Chem. 81, 6165-6174.

Soga, T., Ueno, Y., Naraoka, H., Ohashi, Y., Tomita, M. and Nishioka, T. (2002) Simultaneous determination of anionic intermediates for Bacillus subtilis metabolic pathways by capillary electrophoresis electrospray ionization mass spectrometry. Anal. Chem. 74, 2233-2239.

LEGENDS TO SUPPLEMENTAL FIGURES

S1 Fig. Supplementary data on modulation of *tko*^{25t} phenotype by diet

Time to eclosion of *tko*^{25t} and wild-type flies of sex as shown, grown on media of the indicated composition (see SI for details). In (A) asterisks denote significant differences from flies of the same genotype grown on 0% sucrose medium (Student's *t* test, * showing $p < 0.05$, ** showing $p < 0.01$). For corresponding eclosion data of males see Fig 1A. In (B) and (E), horizontal lines indicate significant differences between flies of a given genotype, grown on different media (Student's *t* test, * showing $p < 0.05$, ** showing $p < 0.01$). For corresponding eclosion data of males grown on high-sugar media, see Fig 4D. In (C) asterisks (**) denote significant difference from flies of the same genotype grown on all other media tested (Student's *t* test, $p < 0.01$), which were not significantly different from each other. For corresponding eclosion data of males see Fig 1B. In (D) there were no significant differences from flies of the same genotype, grown on other media (Student's *t* test, $p > 0.05$). In all experiments eclosion times for *tko*^{25t} flies were also significantly different from those of wild-type flies grown on the same medium (Student's *t* test, $p < 0.01$). For corresponding eclosion data of males see Figs 1 and 4D.

S2 Fig. Supplementary data on the 'anti-sugar' response of *tko*^{25t} flies

Expression levels of various genes, based on QRTPCR, in adult females of the indicated genotypes, grown on high-sugar medium. (A) Malpighian tubule-specific sugar transporters, (B) gut-specific α-glucosidases. All signals normalized to the levels in wild-type females. Horizontal bars denote values significantly different between genotypes (Student's *t* test, * indicating $p < 0.05$, ** indicating $p < 0.01$).

S3 Fig. Supplementary data on metabolite levels in *tko*^{25t} and wild-type flies

Relative levels of different metabolites in adult females or L3 larvae (as shown) of the indicated genotypes and growth conditions, based on (A) findings from enzyme-liked assays, (B) fluorescence spectrometry or (C, D) mass spectrometry. Absolute values are shown for (C) amino acids. Values in (A, B) are normalized to those for wild-type larvae grown on ZS medium, enabling them to be plotted alongside for comparison. A similar plot for those amino acids exhibiting substantial changes (here boxed in red) is shown in Fig 3D. Values in (D) for polyamines are normalized to the level of putrescine in wild-type larvae grown on ZS medium, enabling them to be plotted alongside for comparison. Absolute values from mass spectrometry are given in Table S1. Horizontal bars denote significantly different data classes (Student's *t* test, $p < 0.05$), except in (C), where significant differences in amino acid levels between wild-type and tko^{25t} are shown in Fig 3D, and presented in full in S7 Table.

S4 Fig. Supplementary indicative data on dietary modulation of tko^{25t} phenotype

(A) Time to eclosion of female flies of the indicated genotypes and dietary conditions, on medium supplemented with pyruvate (pyr) or lactate (lact). In the presence of either supplement there were no significant difference in eclosion timing between tko^{25t} flies grown on high-sugar versus zero-sugar medium (Student's *t* test, $p > 0.05$). See also Fig 4A. (B) Summary diagram of the major NADPH-producing enzymes. (C) Activities of the major NADPH-producing enzymes in extracts from *Drosophila* L3 larvae of the indicated genotypes and dietary conditions. (D, E) Time to eclosion of female flies of the indicated genotypes and dietary conditions, on medium supplemented (or not) with ornithine (orn), at the concentrations shown. * denotes value significantly different than for flies of the corresponding genotype and dietary condition, with ornithine *versus* without the supplement (Student's *t* test, $p < 0.05$). (F) Western blots of extracts from L3 larvae of the indicated genotypes and dietary conditions, probed for AMPK, pAMPK (phosphorylated at Thr-172), Akt, pAkt (phosphorylated at Ser-505) or S6K, plus the α-tubulin loading control (αTub). See also Fig. 4F. (G) QRTPCR of mRNAs for four of the *Drosophila* insulin-like peptide (dILP) genes, in larvae of the indicated genotype and dietary condition. Despite the trend, differences

between genotypes were not significant for the dILP genes considered individually (Student's *t* test, $p > 0.05$). (H, I) Analysis of Xbp1 splicing by RTPCR. (H) Agarose gel showing the product fragments diagnostic for the spliced (216S) and unspliced (239U) forms of Xbp1 mRNA (fragment sizes in bp). (I) Analysis by QRTPCR, in larvae of the indicated genotype and dietary condition, revealing only modest differences (all values normalized to those for wild-type larvae grown on high-sugar medium). (J) Time to eclosion of female flies of the indicated genotypes and dietary conditions, on medium supplemented with 12.5 mg/ml dichloroacetate (DCA). Males showed the same trends.

S5 Fig. Supplementary data on effect of cycloheximide and tunicamycin on developmental timing of tko^{25t} and wild-type flies

(A, B) Repeats of experiment shown in Fig 5A, but using various ranges of cycloheximide concentrations. (A) Means $\pm$ SD of times to eclosion of flies of the sex and genotypes indicated, on media containing increasing amounts of cycloheximide. Based on pairwise *t* tests, and considering all the flies of a given sex and genotype cultured at a specific drug concentration as a single population, mean eclosion times were significantly different ($p < 0.01$) at different cycloheximide concentrations for tko^{25t} males or females at all doses tested, compared with flies grown on medium without drug, but the values for the different doses of drug tested were not different from each other. For control flies, values at all concentrations were significantly different from those without drug and from each other, except for 25 *versus* 75 µg/ml. (B) Means $\pm$ SD of times to eclosion of flies of the sex and genotypes indicated, on high-sugar medium, with or without cycloheximide (50 µg/ml). Based on pairwise *t* tests, and considering all the flies of a given sex and genotype cultured at a specific drug concentration as a single population, eclosion timed for flies cultured without drug were significantly different from those cultured with drug in each case ($p < 0.01$). (C) Pooled eclosion data from four independent experiments conducted with different concentration ranges of cycloheximide. Male developmental delay showed consistent decrease with increasing

cycloheximide concentration. Females showed the same trend (Fig 5B). (D) Bang-sensitivity (recovery times) of *tko*^{25t} flies of the sexes indicated, grown on high-sugar medium with or without cycloheximide (150 μg/ml). Wild-type flies were not bang-sensitive. (E) Means $\pm$ SD of times to eclosion of flies of the sex and genotypes indicated, on high-sugar medium, with or without tunicamycin (12 μM). Asterisks denote significant differences between flies of a given sex and genotype cultured with or without drug ($p < 0.01$). A repeat experiment gave the same result.

S6 Fig. Verification that Kyoto GAL4 driver line 113094 directs expression specifically in the gut

Micrographs of dissected L3 larvae expressing GFP driven by Kyoto GAL4 line 113094 (‘gut-GAL4)’, full genotypes as indicated, left-hand panels in visible light, right-hand panels showing green fluorescence. (A) nuclear-localized Stinger-GFP, (B) membrane-localized mCD8-GFP, (C) portion of top image from (B) at higher magnification, to show more detail of structures. As arrowed, GFP is expressed in the salivary glands (sg), gastric caecae (gc), foregut and mid-gut (mg), most strongly in its distal portion, but not in the imaginal discs (id), brain (b), hind-gut (hg), Malpighian tubule (mt), fat body (fb), proventriculus (p), or carcass (c). [Faint signal in carcass is background auto-fluorescence].
