# Supplementary material for: Mitochondrial Dysfunction Plus High-Sugar Diet Provokes a Metabolic Crisis That Inhibits Growth
Source: PLoS One. 2016 Jan 26;11(1):e0145836. doi: 10.1371/journal.pone.0145836 (PMC4728084; doi:10.1371/journal.pone.0145836)
Supplement: S5 Table — (DOC) [file pone.0145836.s012.doc]

**S5 TABLE** – Gene ontology analysis of transcriptomic data

| **Up-regulated on ZS vs. HS in wild-type** | | | |  |  |  | |  | |  | | | |  |
| --- | --- | --- | --- | --- | --- | --- | --- | --- | --- | --- | --- | --- | --- | --- |
| **KEGG term** |  |  |  |  |  | | **Count** | |  | | **Biological process term** | **Count** |  | |
| 00903:Limonene and pinene degradation | | | | |  | | 27 | |  | | GO:0042742~defense response to bacterium | 23 |  | |
| 04142:Lysosome | |  |  |  |  | | 25 | |  | | GO:0006022~aminoglycan metabolic process | 33 |  | |
| 00511:Other glycan degradation | | | |  |  | | 11 | |  | | GO:0006508~proteolysis | 89 |  | |
| 00600:Sphingolipid metabolism | | | |  |  | | 10 | |  | | GO:0019731~antibacterial humoral response | 13 |  | |
| 00980:Metabolism of xenobiotics by cytochrome P450 | | | | | | | 16 | |  | | GO:0006030~chitin metabolic process | 26 |  | |
| 00982:Drug metabolism | | |  |  |  | | 16 | |  | | GO:0006026~aminoglycan catabolic process | 11 |  | |
| 00830:Retinol metabolism | | |  |  |  | | 9 | |  | | GO:0000272~polysaccharide catabolic process | 11 |  | |
| 00564:Glycerophospholipid metabolism | | | |  |  | | 12 | |  | | GO:0006643~membrane lipid metabolic process | 9 |  | |
| 00983:Drug metabolism | | |  |  |  | | 11 | |  | | GO:0007559~histolysis | 15 |  | |
| 00500:Starch and sucrose metabolism | | | |  |  | | 11 | |  | | GO:0006027~glycosaminoglycan catabolic process | 7 |  | |
|  |  |  |  |  |  | |  | |  | |  |  |  | |
|  |  |  |  |  |  | |  | |  | |  |  |  | |
| **Up-regulated on ZS vs. HS in *tko*^25t^** | | | |  |  | |  | |  | |  |  |  | |
|  |  |  |  |  |  | |  | |  | |  |  |  | |
| **KEGG term** |  |  |  |  |  | | **Count** | |  | | **Biological process term** | **Count** |  | |
| 00350:Tyrosine metabolism | | |  |  |  | | 12 | |  | | GO:0009072~aromatic amino acid family metabolic process | 11 |  | |
| 00980:Metabolism of xenobiotics by cytochrome P450 | | | | | | | 13 | |  | | GO:0019752~carboxylic acid metabolic process | 27 |  | |
| 00982:Drug metabolism | | |  |  |  | | 13 | |  | | GO:0006520~cellular amino acid metabolic process | 19 |  | |
| 00903:Limonene and pinene degradation | | | | |  | | 14 | |  | | GO:0006508~proteolysis | 46 |  | |
| 00511:Other glycan degradation | | | |  |  | | 6 | |  | | GO:0006570~tyrosine metabolic process | 6 |  | |
| 00260:Glycine, serine and threonine metabolism | | | | |  | | 6 | |  | | GO:0044106~cellular amine metabolic process | 19 |  | |
| 00480:Glutathione metabolism | | | |  |  | | 9 | |  | | GO:0006026~aminoglycan catabolic process | 6 |  | |
| 00380:Tryptophan metabolism | | |  |  |  | | 4 | |  | | GO:0000272~polysaccharide catabolic process | 6 |  | |
| 00830:Retinol metabolism | | |  |  |  | | 5 | |  | | GO:0006022~aminoglycan metabolic process | 12 |  | |
| 00360:Phenylalanine metabolism | | | |  |  | | 4 | |  | | GO:0046653~tetrahydrofolate metabolic process | 3 |  | |

| **Down-regulated on ZS vs. HS in OR** | | | |  |  |  |  |  |
| --- | --- | --- | --- | --- | --- | --- | --- | --- |
|  |  |  |  |  |  |  |  |  |
| **KEGG term** |  |  |  |  | **Count** |  | **Biological process term** | **Count** |
| 00010:Glycolysis / Gluconeogenesis | | | |  | 15 |  | GO:0045333~cellular respiration | 25 |
| 00190:Oxidative phosphorylation | | | |  | 25 |  | GO:0019752~carboxylic acid metabolic process | 40 |
| 00020:Citrate cycle (TCA cycle) | | |  |  | 12 |  | GO:0006006~glucose metabolic process | 17 |
| 00620:Pyruvate metabolism | | |  |  | 12 |  | GO:0006030~chitin metabolic process | 23 |
| 00903:Limonene and pinene degradation | | | | | 16 |  | GO:0006022~aminoglycan metabolic process | 26 |
| 00564:Glycerophospholipid metabolism | | | |  | 13 |  | GO:0044275~cellular carbohydrate catabolic process | 15 |
| 00350:Tyrosine metabolism | | |  |  | 9 |  | GO:0019320~hexose catabolic process | 13 |
| 00360:Phenylalanine metabolism | | | |  | 6 |  | GO:0022904~respiratory electron transport chain | 17 |
| 00030:Pentose phosphate pathway | | | |  | 6 |  | GO:0046365~monosaccharide catabolic process | 13 |
|  |  |  |  |  |  |  |  | 27 |
|  |  |  |  |  |  |  |  |  |
| **Down-regulated on ZS vs. HS in *tko*^25t^** | | | |  |  |  |  |  |
|  |  |  |  |  |  |  |  |  |
| **KEGG term** |  |  |  |  | **Count** |  | **Biological process term** | **Count** |
| 04080:Neuroactive ligand-receptor interaction | | | | | 6 |  | GO:0006508~proteolysis | 59 |
| 00480:Glutathione metabolism | | | |  | 6 |  | GO:0006030~chitin metabolic process | 23 |
| 00903:Limonene and pinene degradation | | | | | 7 |  | GO:0006022~aminoglycan metabolic process | 24 |
| 00982:Drug metabolism | | |  |  | 6 |  | GO:0006820~anion transport | 8 |
| 00010:Glycolysis / Gluconeogenesis | | | |  | 5 |  | GO:0046942~carboxylic acid transport | 8 |
|  |  |  |  |  |  |  | GO:0015711~organic anion transport | 5 |
|  |  |  |  |  |  |  | GO:0006865~amino acid transport | 6 |
|  |  |  |  |  |  |  | GO:0007594~puparial adhesion | 4 |
|  |  |  |  |  |  |  | GO:0015672~monovalent inorganic cation transport | 12 |
|  |  |  |  |  |  |  | GO:0015698~inorganic anion transport | 4 |

| **Up-regulated in *tko*^25t^ vs. OR on HS** | | | |  |  |  |  |  |  |
| --- | --- | --- | --- | --- | --- | --- | --- | --- | --- |
|  |  |  |  |  |  |  |  |  |  |
| **KEGG term** |  |  |  |  |  | **Count** |  | **Biological process term** | **Count** |
| 00230:Purine metabolism | | |  |  |  | 24 |  | GO:0009124~nucleoside monophosphate biosynthetic process | 16 |
| 00500:Starch and sucrose metabolism | | | |  |  | 12 |  | GO:0006022~aminoglycan metabolic process | 28 |
| 00010:Glycolysis / Gluconeogenesis | | | |  |  | 11 |  | GO:0019731~antibacterial humoral response | 10 |
| 00030:Pentose phosphate pathway | | | |  |  | 7 |  | GO:0042742~defense response to bacterium | 16 |
| 00051:Fructose and mannose metabolism | | | | |  | 7 |  | GO:0006030~chitin metabolic process | 22 |
| 00511:Other glycan degradation | | | |  |  | 5 |  | GO:0034654~nucleobase, nucleoside, nucleotide and NA biosynthesis | 27 |
| 00980:Metabolism of xenobiotics by cytochrome P450 | | | | | | 10 |  | GO:0034404~nucleobase, nucleoside and nucleotide biosynthesis | 27 |
| 00620:Pyruvate metabolism | | |  |  |  | 8 |  | GO:0006508~proteolysis | 86 |
| 00982:Drug metabolism | | |  |  |  | 10 |  | GO:0019320~hexose catabolic process | 11 |
| 00790:Folate biosynthesis | | |  |  |  | 5 |  | GO:0044275~cellular carbohydrate catabolic process | 12 |
|  |  |  |  |  |  |  |  |  |  |
|  |  |  |  |  |  |  |  |  |  |
| **Up-regulated in *tko*^25t^ vs. OR on ZS** | | | |  |  |  |  |  |  |
|  |  |  |  |  |  |  |  |  |  |
| **KEGG term** |  |  |  |  |  | **Count** |  | **Biological process term** | **Count** |
| 00350:Tyrosine metabolism | | |  |  |  | 10 |  | GO:0006508~proteolysis | 87 |
| 00561:Glycerolipid metabolism | | | |  |  | 10 |  | GO:0019752~carboxylic acid metabolic process | 38 |
| 00903:Limonene and pinene degradation | | | | |  | 14 |  | GO:0009063~cellular amino acid catabolic process | 10 |
| 00620:Pyruvate metabolism | | |  |  |  | 9 |  | GO:0009309~amine biosynthetic process | 12 |
| 00051:Fructose and mannose metabolism | | | | |  | 7 |  | GO:0006022~aminoglycan metabolic process | 23 |
| 00980:Metabolism of xenobiotics by cytochrome P450 | | | | | | 10 |  | GO:0046394~carboxylic acid biosynthetic process | 14 |
| 00982:Drug metabolism | | |  |  |  | 10 |  | GO:0009310~amine catabolic process | 10 |
| 00010:Glycolysis / Gluconeogenesis | | | |  |  | 8 |  | GO:0046395~carboxylic acid catabolic process | 11 |
| 00360:Phenylalanine metabolism | | | |  |  | 5 |  | GO:0006030~chitin metabolic process | 19 |
| 00910:Nitrogen metabolism | | |  |  |  | 5 |  | GO:0044106~cellular amine metabolic process | 24 |

| **Down-regulated in *tko*^25t^ vs. OR on HS** | | | |  |  |  |  |  |
| --- | --- | --- | --- | --- | --- | --- | --- | --- |
|  |  |  |  |  |  |  |  |  |
| **KEGG term** |  |  |  |  | **Count** |  | **Biological process term** | **Count** |
| 00190:Oxidative phosphorylation | | | |  | 56 |  | GO:0006119~oxidative phosphorylation | 54 |
| 00260:Glycine, serine and threonine metabolism | | | | | 15 |  | GO:0043436~oxoacid metabolic process | 78 |
| 00903:Limonene and pinene degradation | | | | | 33 |  | GO:0022900~electron transport chain | 39 |
| 00350:Tyrosine metabolism | | |  |  | 18 |  | GO:0022904~respiratory electron transport chain | 33 |
| 00280:Valine, leucine and isoleucine degradation | | | | | 17 |  | GO:0015980~energy derivation by oxidation of organic compounds | 41 |
| 00980:Metabolism of xenobiotics by cytochrome P450 | | | | | 26 |  | GO:0006520~cellular amino acid metabolic process | 42 |
| 00071:Fatty acid metabolism | | |  |  | 18 |  | GO:0044106~cellular amine metabolic process | 45 |
| 00360:Phenylalanine metabolism | | | |  | 13 |  | GO:0006022~aminoglycan metabolic process | 39 |
| 00982:Drug metabolism | | |  |  | 26 |  | GO:0055086~nucleobase, nucleoside and nucleotide metabolic process | 49 |
| 00010:Glycolysis / Gluconeogenesis | | | |  | 21 |  | GO:0006732~coenzyme metabolic process | 29 |
|  |  |  |  |  |  |  |  |  |
|  |  |  |  |  |  |  |  |  |
| **Down-regulated in *tko*^25t^ vs. OR on ZS** | | | |  |  |  |  |  |
|  |  |  |  |  |  |  |  |  |
| **KEGG term** |  |  |  |  | **Count** |  | **Biological process term** | **Count** |
| 00903:Limonene and pinene degradation | | | | | 36 |  | GO:0006508~proteolysis | 144 |
| 00980:Metabolism of xenobiotics by cytochrome P450 | | | | | 28 |  | GO:0006022~aminoglycan metabolic process | 35 |
| 00982:Drug metabolism | | |  |  | 28 |  | GO:0006030~chitin metabolic process | 30 |
| 00830:Retinol metabolism | | |  |  | 17 |  | GO:0019752~carboxylic acid metabolic process | 46 |
| 00983:Drug metabolism | | |  |  | 22 |  | GO:0042742~defense response to bacterium | 18 |
| 00511:Other glycan degradation | | | |  | 11 |  | GO:0044242~cellular lipid catabolic process | 10 |
| 04080:Neuroactive ligand-receptor interaction | | | | | 15 |  | GO:0006026~aminoglycan catabolic process | 11 |
| 00053:Ascorbate and aldarate metabolism | | | | | 14 |  | GO:0000272~polysaccharide catabolic process | 11 |
| 04142:Lysosome | |  |  |  | 25 |  | GO:0006631~fatty acid metabolic process | 14 |
| 00150:Androgen and estrogen metabolism | | | | | 13 |  | GO:0034754~cellular hormone metabolic process | 7 |
